# Anti-inflammatory properties and characterization of water extracts obtained from *Callicarpa kwangtungensis* Chun using in vitro and in vivo rat models

**DOI:** 10.1038/s41598-024-61892-9

**Published:** 2024-05-14

**Authors:** Jun-Jian Li, Li Li, Shan-Shan Su, Mei-Lan Liao, Qiu-Zi Gong, Mei Liu, Shan Jiang, Zai-Qi Zhang, Hua Zhou, Jian-Xin Liu

**Affiliations:** 1 School of Pharmaceutical Sciences, School of Basic Medical Sciences, Hunan Provincial Key Laboratory of Dong Medicine, Hunan University of Medicine, Huaihua, China; 2 School of Pharmacy, Heilongjiang University of Chinese Medicine, Harbin, Heilongjiang China; 3 Guangdong Provincial Hospital of Chinese Medicine, Guangdong-Hong Kong-Macau Joint Lab On Chinese Medicine and Immune Disease Research, State Key Laboratory of Dampness Syndrome of Chinese Medicine, Guangdong Provincial Academy of Chinese Medical Sciences, Second Affiliated Hospital of Gzangzhou University of Chinese Medicine, Guangzhou, Guangdong China; 4 School of Pharmaceutical Science, University of South China, Hengyang, China

**Keywords:** Biochemistry, Drug discovery, Immunology

## Abstract

*Callicarpa kwangtungensis* Chun (CK) is a common remedy exhibits anti-inflammatory properties and has been used in Chinese herbal formulations, such as KangGongYan tablets. It is the main component of KangGongYan tablets, which has been used to treat chronic cervicitis caused by damp heat, red and white bands, cervical erosion, and bleeding. However, the anti-inflammatory effects of CK water extract remains unknown. This study assessed the anti-inflammatory effects of CK in vivo and in vitro, characterized its main components in the serum of rats and verified the anti-inflammatory effects of serum containing CK. Nitric oxide (NO), tumour necrosis factor *α* (TNF-α) and interleukin-6 (IL-6) release by RAW264.7 cells was examined by ELISA and Griess reagents. Inflammation-related protein expression in LPS-stimulated RAW264.7 cells was measured by western blotting. Furthermore, rat model of foot swelling induced by λ-carrageenan and a collagen-induced arthritis (CIA) rat model were used to explore the anti-inflammatory effects of CK. The components of CK were characterized by LC–MS, and the effects of CK-containing serum on proinflammatory factors levels and the expression of inflammation-related proteins were examined by ELISA, Griess reagents and Western blotting. CK suppressed IL-6, TNF-α, and NO production, and iNOS protein expression in LPS-stimulated RAW264.7 cells. Mechanistic studies showed that CK inhibited the phosphorylation of ERK, P38 and JNK in the MAPK signaling pathway, promoted the expression of IκBα in the NF-κB signaling pathway, and subsequently inhibited the expression of iNOS, thereby exerting anti-inflammatory effects. Moreover, CK reduced the swelling rates with λ-carrageenan induced foot swelling, and reduced the arthritis score and incidence in the collagen-induced arthritis (CIA) rat model. A total of 68 compounds in CK water extract and 31 components in rat serum after intragastric administration of CK were characterized. Serum pharmacological analysis showed that CK-containing serum suppressed iNOS protein expression and NO, TNF-α, and IL-6 release. CK may be an anti-inflammatory agent with therapeutic potential for acute and chronic inflammatory diseases, especially inflammatory diseases associated with MAPK activation.

## Introduction

Inflammatory cascades are automatic defence responses in the human body^1^ and are the only identified mechanism for the restoration of tissue after injury. Thus, they play pivotal roles in the immune system when infection and injury occur^2^. However, excessive and chronic inflammation can damage various tissues in the human body^3^, resulting in the initiation and progression of diseases, including cancer, rheumatoid arthritis (RA), neurodegenerative diseases and other immune diseases^4^. Currently, glucocorticoids and nonsteroidal drugs are widely used to treat RA in clinical practice^5^. However, these medicines have various side effects, including gastrointestinal reactions and increasing cardiovascular risk^6^.

Rheumatoid arthritis (RA) is systemic inflammatory response caused by immune system overactivation^7^. Its main pathological features are cell proliferation, microvascular proliferation^8^, inflammatory cell infiltration, and synovial tissue proliferation^9^. Although RA is a common chronic-inflammatory disease in the clinic and the therapeutic strategies for RA are continually improving, there is a lack of specific medicines with low toxicity and high efficiency for treating this disease^10–12^. Therefore, developing drugs for treating RA is a priority.

Macrophages play a pivotal role in inflammatory progression, and can be activated by multiple irritants such as lipopolysaccharide (LPS)^13^. The progression of inflammation can be promoted by increases in nitric oxide (NO), tumour necrosis factor α (TNF-α) and interleukin-6 (IL-6) in activated macrophages.

Nuclear factor kappa-B (NF-κB) and mitogen-activated protein kinase (MAPK) are vital intracellular signaling pathways. The transcription factor NF-κB is a major regulator of inflammatory responses^14^. Nuclear translocation of active p65 triggers an LPS-induced inflammatory response in macrophages. MAPK can regulate TNF-α and IL-6 expression and promote inducible nitric oxide synthase (iNOS) and cyclooxygenase-2 (COX-2) expression after being activated by stimuli such as LPS^15,16^. Therefore, inhibiting NF-κB and MAPK activation is critical for anti-inflammatory strategies.

Investigating the anti-inflammatory effect of natural ingredients in traditional Chinese medicine has proven to be useful and productive and traditional Chinese medicine for treating RA has also attracted much attention^17,18^. However, the ingredients of traditional Chinese medicine are very complex. Serum pharmaco-chemistry is an effective method that identifies components found in serum after intragastric administration and examines the activity of medicated serum. It is commonly used to investigate the effective ingredients and pharmacological activity of traditional Chinese medicine^19^.

*Callicarpa kwangtungensis* Chun can be used to treat pain^20^. Phenylethanol glycosides, flavonoids, and terpenes are the main chemical components of CK. Phenylethanol glycoside is one of the main active ingredients with the highest levels. Forsythiaside B and poliumoside are phenylethanol glycosides, and their total levels are quality control standards for CK in the Pharmacopoeia of the People’s Republic of China^21^. Pharmacological studies have shown that CK represses the early exudation of experimental inflammation. However, the anti-inflammatory components and potential of CK remain unknown. In this study, the anti-inflammatory properties and components of CK in serum were explored for the first time and the molecular mechanism was investigated.

## Results

### CK suppressed iNOS protein but not COX-2 protein expression in LPS-induced RAW264.7 cells

**Figure 1:**
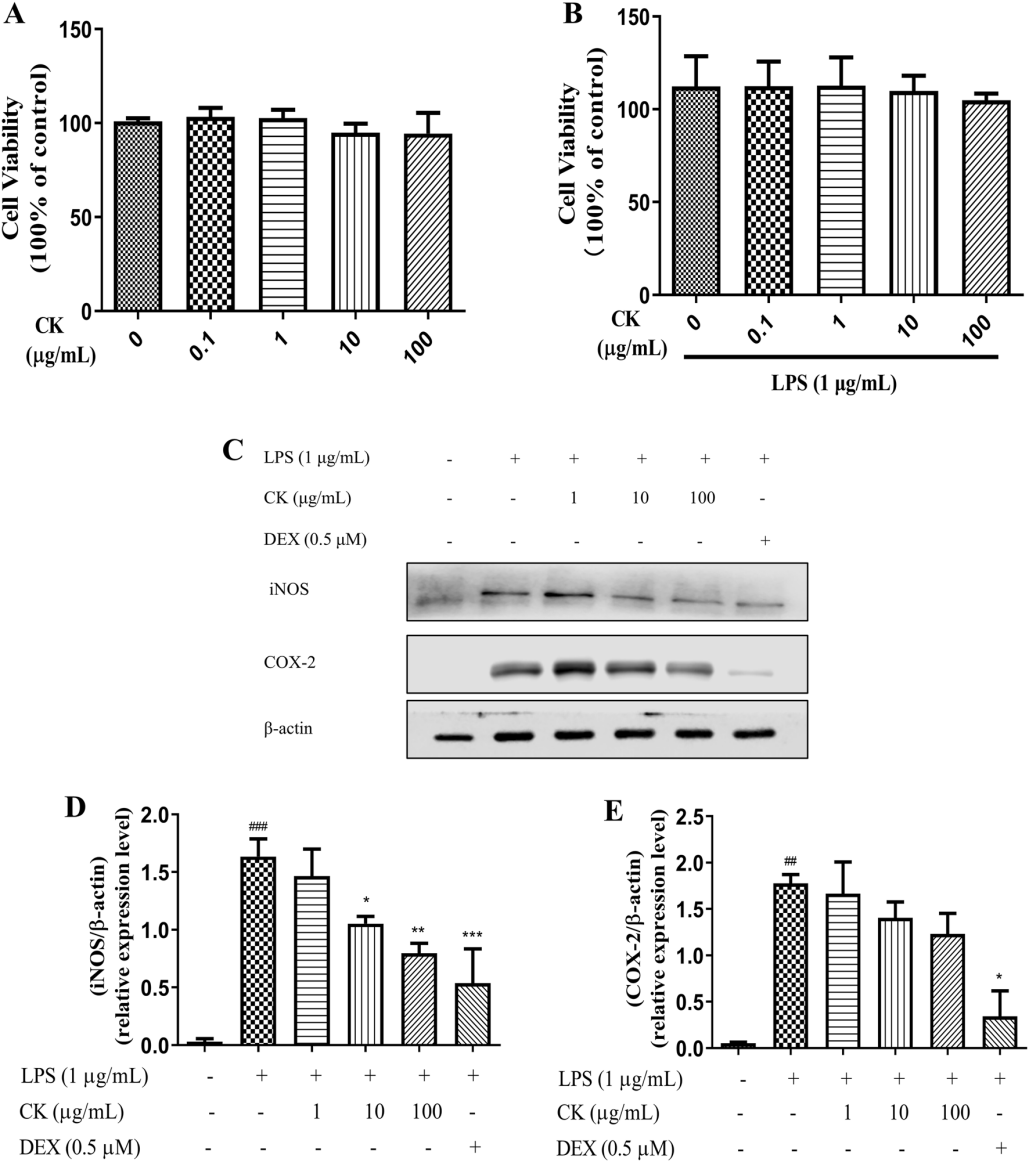
CK suppressed the expression of iNOS in LPS-stimulated RAW264.7 cells. (**A** and **B**) Effect of CK on the viability of RAW264.7 cells. (**C**–**E**) Effect of CK on the expression of COX-2 and iNOS (Due to the blurring of marker, the representative image of iNOS is replaced after the editor’s consent. All the complete strip images in this article are in the supplementary file.) in LPS-stimulated RAW264.7 cells. Cells were pretreated with CK (1, 10, or 100 µg/mL) or DEX for 1 h and then stimulated with LPS (1 µg/mL) for 18 h. The protein expression of iNOS and COX-2 was analysed by western blotting. The data are presented as the means ± SDs of three experiments. ^##^*P* < 0.01, ^###^*P* < 0.001, versus the control group. ^*^P < 0.05, ^**^P < 0.01, ^***^P < 0.001, versus the LPS-stimulated group.

The results of the MTT assay showed that CK concentrations less than 100 µg/mL had no effect on the viability of macrophages treated with or without LPS (Fig. 1A and B). Therefore, 1, 10 and 100 µg/mL CK were used to explore the anti-inflammatory effects of CK water extract on LPS-induced RAW264.7 cells. After 18 h of LPS stimulation, iNOS and COX-2 expression was strongly increased (Fig. 1C–E). Compared with that in the LPS stimulation group, the protein level of iNOS and COX-2 in the DEX group significantly decreased. The protein level of iNOS in the CK group decreased in a concentration-dependent manner (Fig. 1D). However, CK did not markedly decrease COX-2 protein expression (Fig. 1E).

### CK reduced NO, IL-6, and TNF-α production in LPS-stimulated RAW264.7 cells

**Figure 2:**
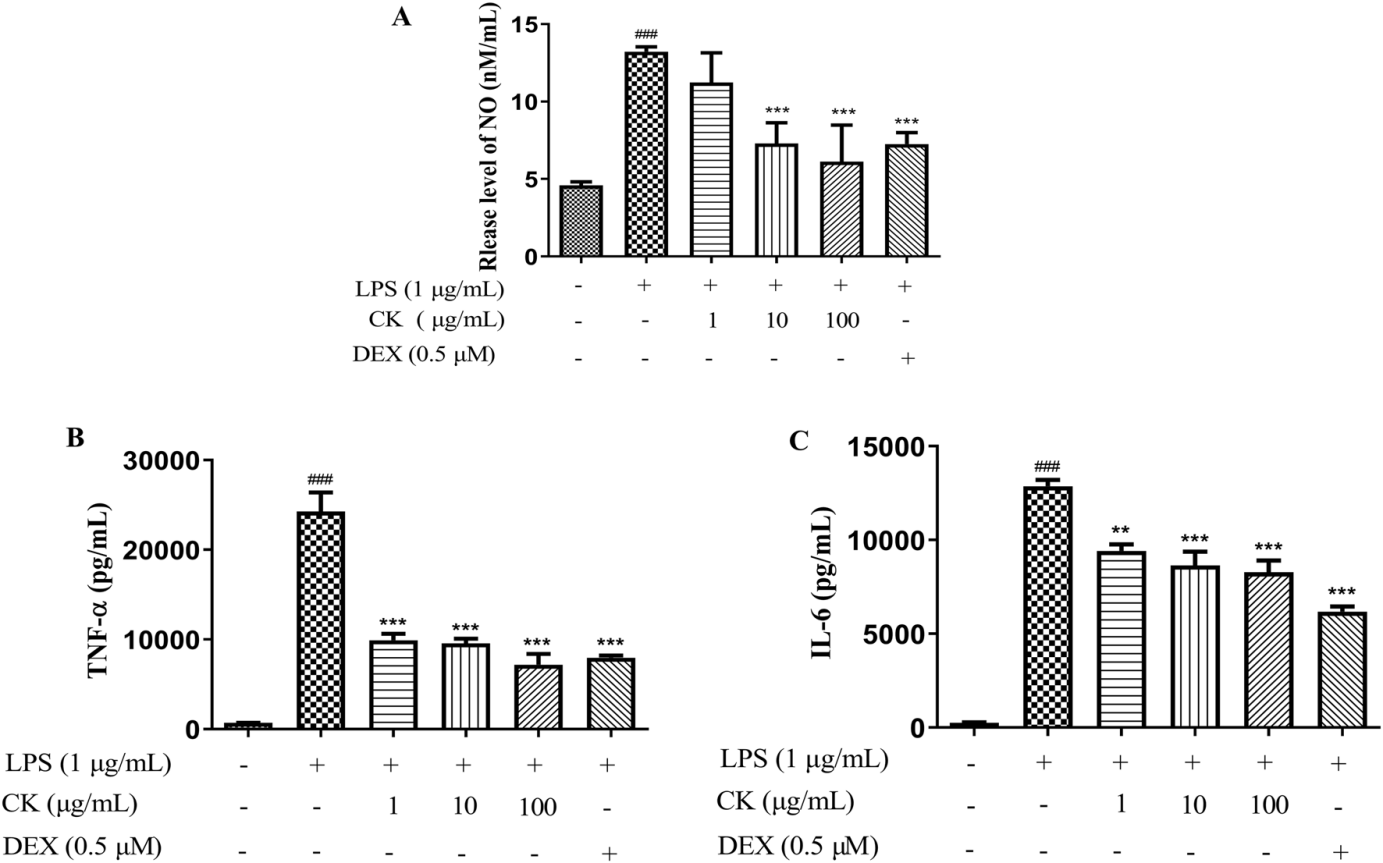
Effect of CK on NO and inflammatory cytokine production in LPS-stimulated RAW264.7 cells. The release of NO into the culture supernatant was examined with a Griess reagent kit (**A**). The levels of TNF-α (**B**) and IL-6 (**C**) were determined using ELISA kits. The values are expressed as means ± SDs (n = 3). ^###^*P* < 0.001, versus the control group. ^*^P < 0.05, ^**^P < 0.01, ^***^P < 0.001, versus the LPS-stimulated group.

NO, which is a signaling molecule, can affect inflammatory progression. TNF-α can mediate acute and chronic inflammation^22^ and stimulate the synthesis of IL-6, which maintains the inflammatory response through cytokines with overlapping capabilities. Compared with that in the LPS stimulation group, the NO, IL-6, and TNF-α production in the DEX group significantly decreased. The CK extract reduced the release of NO in the medium–high concentration group (Fig. 2A) and decreased the levels of these inflammatory cytokines at concentrations of 1, 10, and 100 µg/mL (Fig. 2B and C). The results showed that the CK extract inhibited LPS-induced inflammatory damage in RAW264.7 cells.

### CK increased IκBα expression in LPS-stimulated RAW264.7 cells

**Figure 3:**
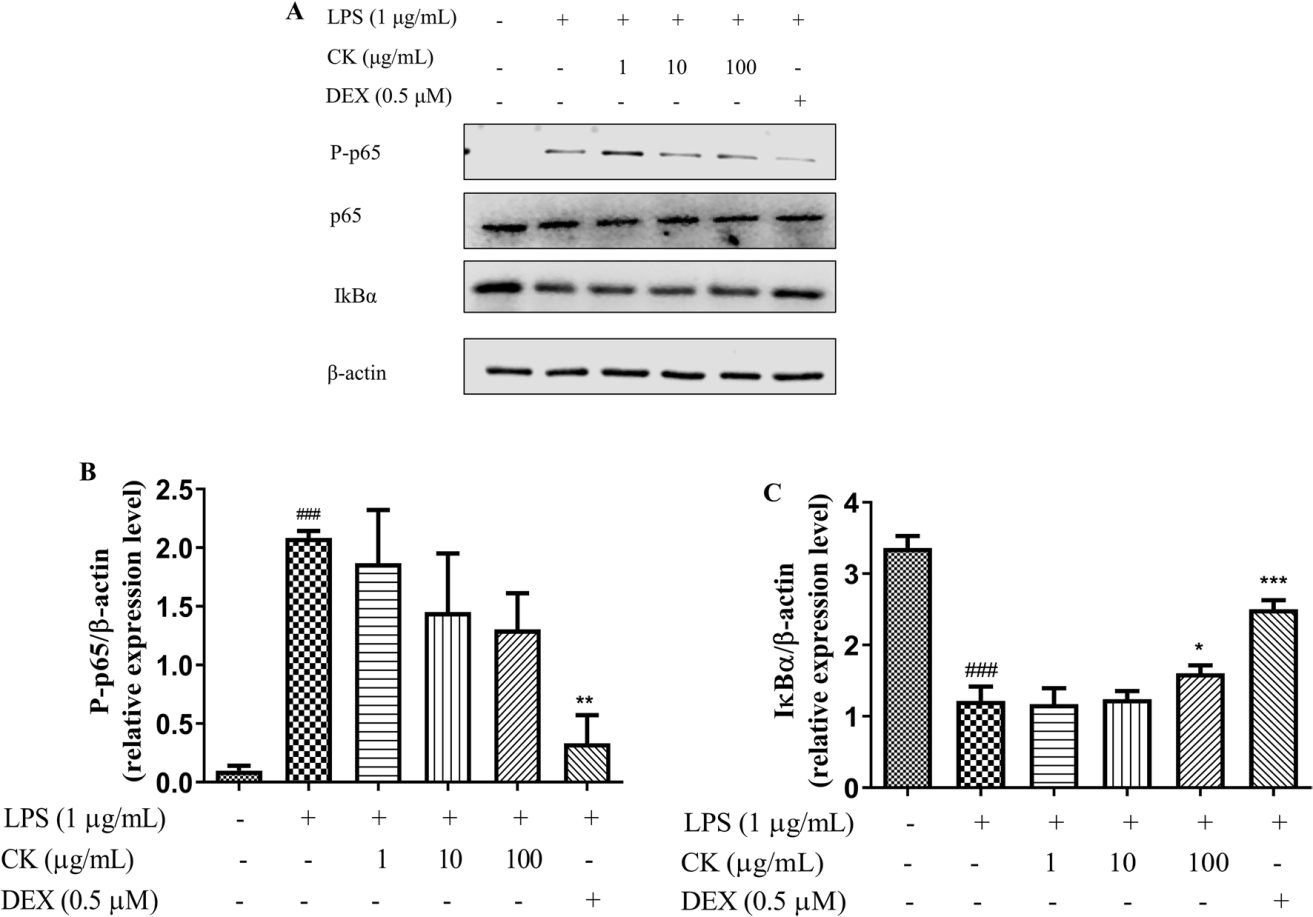
Effect of CK on the NF-κB signaling pathway. Total cell proteins were prepared, the expression (Due to the blurring of marker, the representative image of p65 is replaced after the editor’s consent. All the complete strip images in this article are in the supplementary file.) and phosphorylation levels of p65 (**A**, **B**), and IκBα expression (**C**) were examined by western blotting. The results are presented as an average of ± SD from three independent experiments. ^###^*P* < 0.001, versus the control group. ^**^P < 0.01, versus the LPS-stimulated group.

NF-κB, which is an important nuclear transcription factor^23^, can regulate the inflammatory response and immune response^14^, and is related to rheumatoid arthritis^24^. To gain further insights into how CK inhibits inflammation, LPS-induced RAW264.7 cells were used to investigate the effect of CK on the NF-κB pathway. After 15 min of LPS stimulation, the phosphorylation of p65 increased and IκBα expression reduced. Compared with that in the LPS stimulation group, the phosphorylation of p65 in the DEX group reduced and IκBα expression in the DEX group increased. The decrease of IκBα expression by LPS stimulation was inhibited by CK. However, p65 phosphorylation was not significantly inhibited after treatment with the CK water extract (Fig. 3).

### CK suppressed MAPK activation in LPS-stimulated RAW264.7 cells

**Figure 4:**
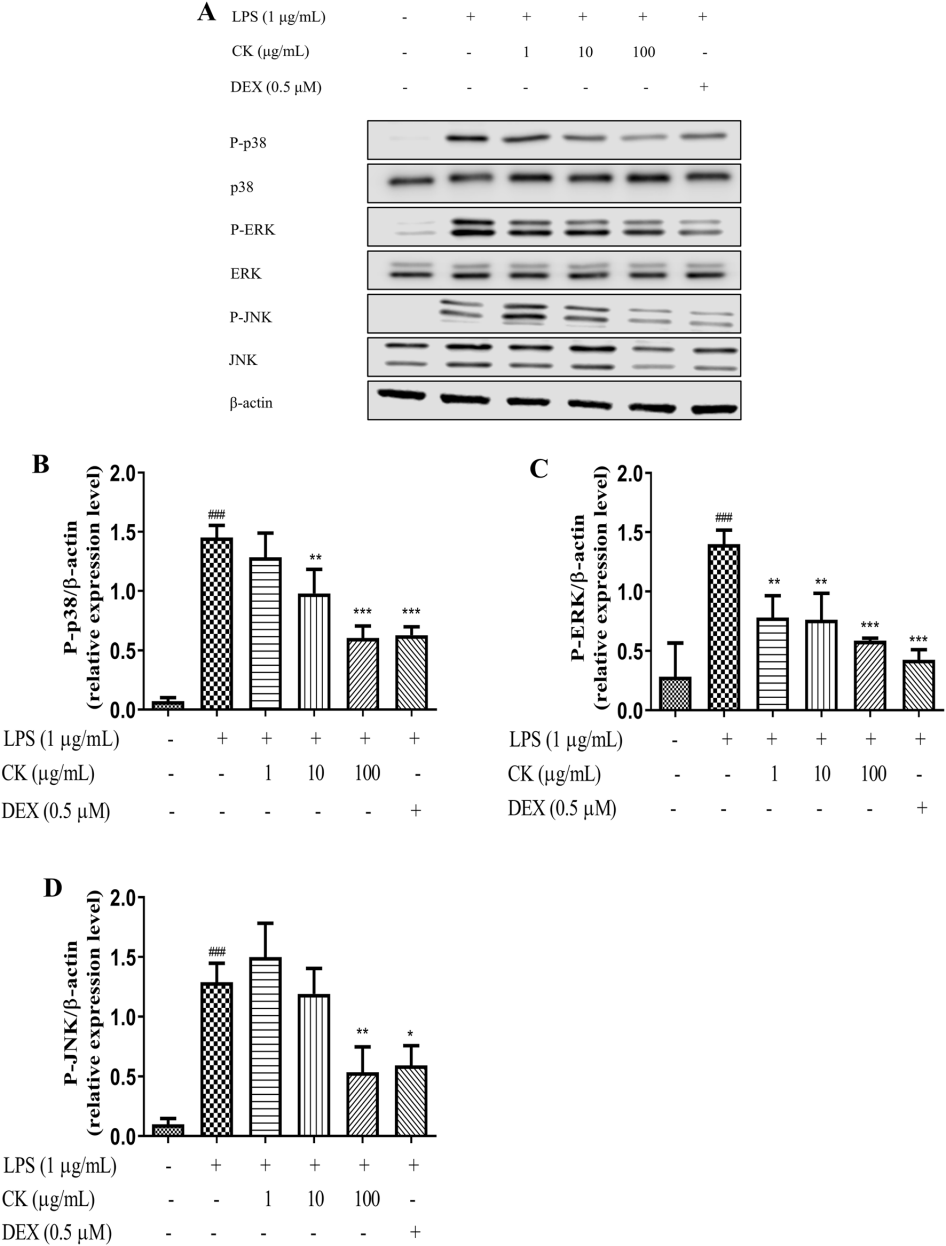
Effect of CK on the MAPK signaling pathway. The cells were plated in 12-well plates, incubated for 24 h, incubated with CK or DEX for 1 h, and then stimulated for 15 min with LPS (1 µg/mL). Total cell proteins were prepared and the expression (**A**) and phosphorylation levels of p38 (**B**), ERK (**C**) and JNK (**D**) were examined by western blotting. The results are presented as the average ± SD from three independent experiments. ^###^*P* < 0.001, versus the control group. ^*^P < 0.05, ^**^P < 0.01, ^***^P < 0.001, versus the LPS-stimulated group.

MAPKs can regulate various physiological processes^25^ and are involved in TNF-α and IL-6 productions, as well as iNOS and COX-2 protein expression^26^. Therefore, MAPK-associated proteins were examined by western blot analysis to investigate the molecular mechanism of the anti-inflammatory effect of CK. Our results showed that the JNK, p38 and EPK phosphorylation in LPS-induced RAW264.7 cells (Fig. 4) were significantly inhibited by CK or DEX.

### CK alleviated foot swelling in a λ-carrageenan induced rat foot swelling model

**Figure 5:**
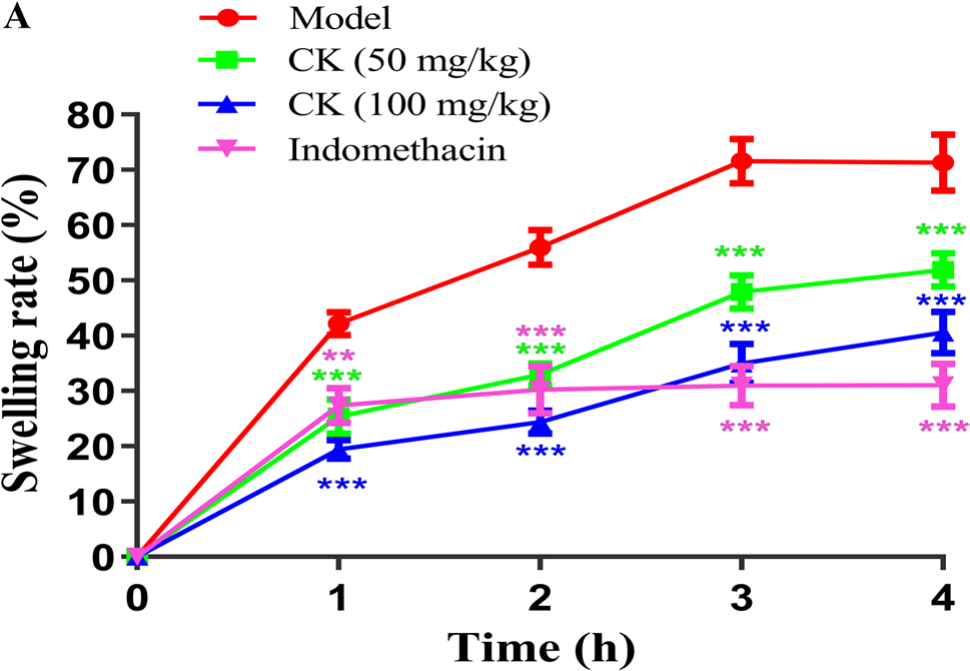
Effect of CK on foot swelling induced by λ-carrageenan in rats. (**A**) The foot swelling rates (%) of rats (n = 10) treated with carrageenan at 1 h, 2 h, 3 h and 4 h showed the difference in paw volume between the model group and the treatment group. ^**^P < 0.01, ^***^P < 0.001, versus the model group.

λ-Carrageenan is a classic inflammatory inducer, and the resulting inflammatory response is maintained by the release of prostaglandins and nitric oxide^27^. Thus, a certain dose of λ-carrageenan was subcutaneously injected into the toes or ankles of rats to cause toe and joint swelling. We evaluated the efficacy of the CK extract by measuring the volume toe swelling and comparing the swelling rate of the toe or ankle joint. Our results showed that CK markedly decreased the amount of oedema in the paws of the rats (Fig. 5).

### CK reduced the arthritis score and incidence of arthritis in a collagen-induced arthritis rat model

**Figure 6:**
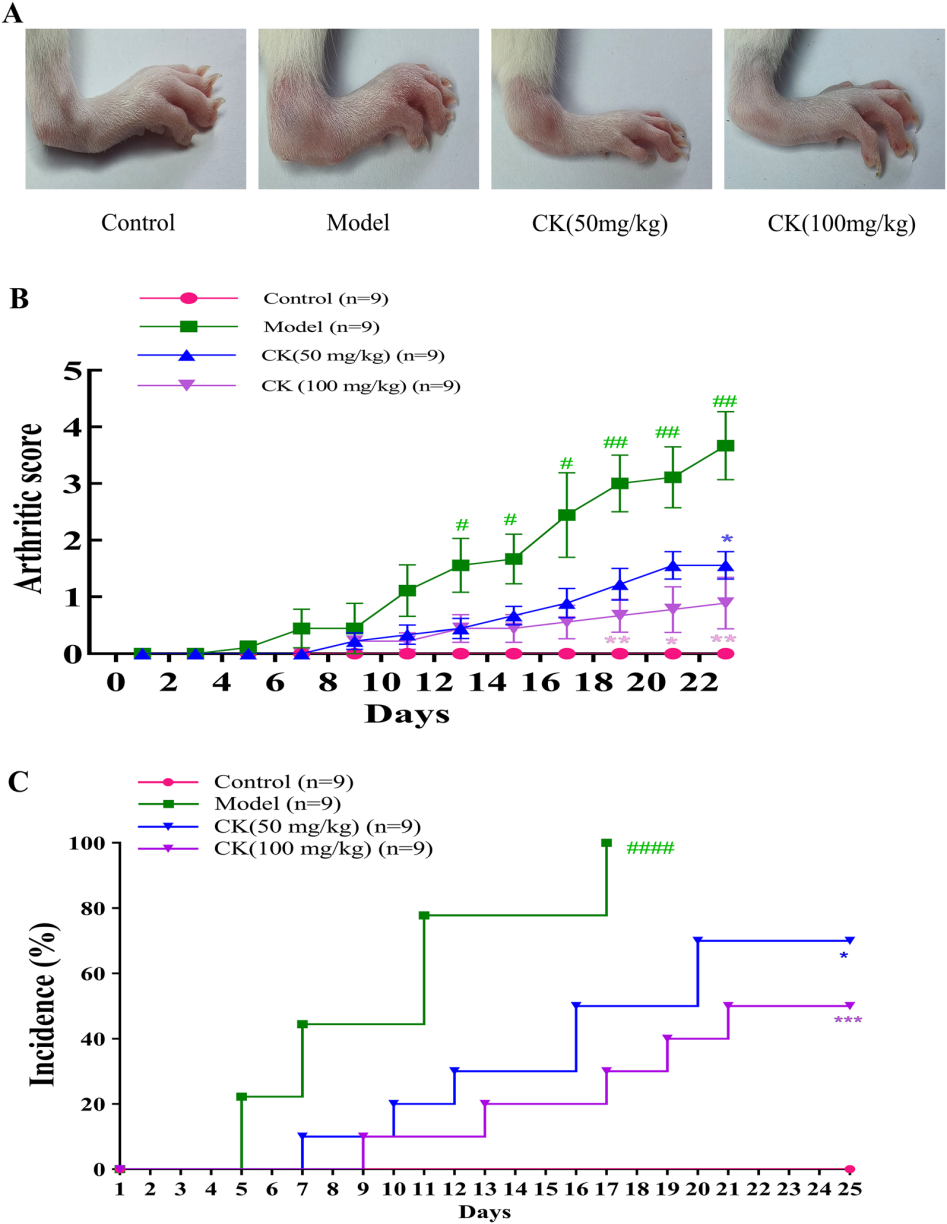
Effect of CK on a collagen-induced arthritis (CIA) rat model. (**A**) Representative images of the effect of CK on CIA rats. (**B**) Effect of CK on the arthritis score. (**C**) Effect of CK on the incidence. The results are presented as the average ± SD (n = 9). ^#^*P* < 0.05, ^##^*P* < 0.01, ^####^*P* < 0.0001 versus the control group. ^*^P < 0.05, ^**^P < 0.01, ^***^P < 0.001, versus the model group.

The histological and immunological changes in the rat CIA model are highly similar to those in human RA^28,29^. Therefore, we used a CIA rat model to examine the therapeutic effect of CK^30^. The arthritis score is an important index reflecting the progression of arthritis^31^. The control group did not show any symptoms; therefore, the score was 0. Collagen-induced rats showed obvious arthritis symptoms, and the score increased with time. Compared with those in the model group, the arthritis symptoms of rats treated with CK (100 mg/kg) were significantly alleviated, and the arthritis score and incidence of arthritis were significantly reduced (Fig. 6A–C).

### Anti-inflammatory components of CK

**Figure 7:**
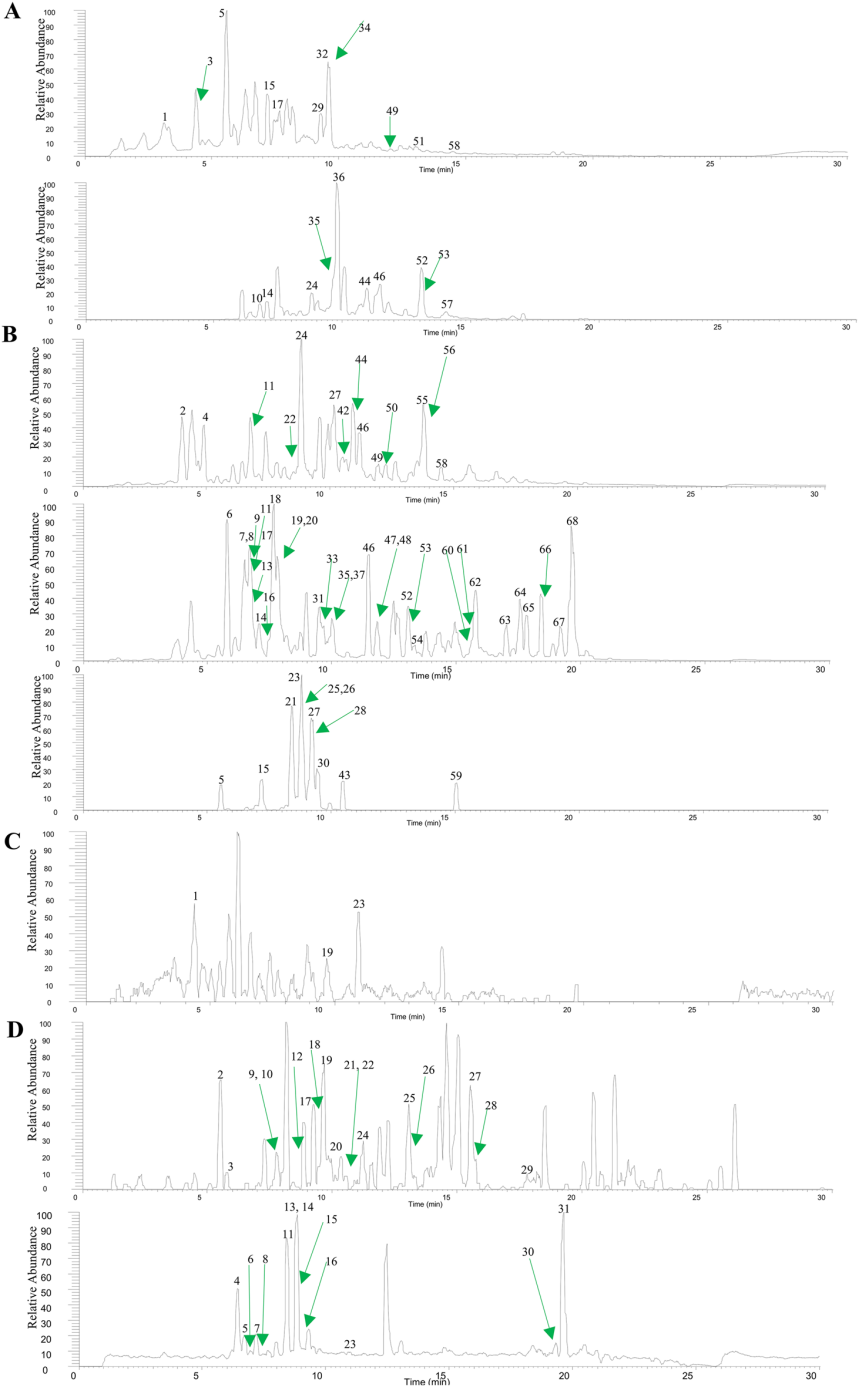
The components of CK were verified by UHPLC-Q-Exactive Orbitrap MS. (**A**) Positive ionization-mode analysis of the CK water extract. (**B**) Negative ionization mode-analysis of CK water extract. (**C**) Positive ionization-mode analysis of CK-containing serum. (**D**) Negative ionisation mode-analysis of CK-containing serum. The number indicates the chemical composition. The arrow points to the peak time of each chemical component.

UHPLC-Q-Exactive Orbitrap MS was used to analyse the CK water extract and medicated serum. A total of 68 components in the CK water extract were preliminarily characterized (Table 1). Moreover, we performed a preliminary analysis of the 31 migrating constituents found in rat serum (Table 2). The compounds were identified based on qualitative analysis and comparison to published literature^32^. The total ion chromatogram is presented in Fig. 7.

**Table 1:** Identification of 68 compounds from *Callicarpa kwangtungensis* Chun water extract.

No	T_R_	Identification	Category	Formula	Ion mode	Theo. Mass (m/z)	Obser. Mass (m/z)	Error (ppm)
1	3.12	Coniferaldehyde	Phenols	C_10_H_10_O_3_	[M + H]^+^	179.07027	179.07021	− 0.339
2	4.01	2-hydroxyl-5-methoxybenzoic acid	Organic acids	C_8_H_8_O_4_	[M − H]^−^	167.03498	167.03362	− 1.588
3	4.37	3,4,5-trimethoxyphenyl-β-D-glucopyranoside	Phenols	C_15_H_22_O_9_	[M + H]^+^	347.13366	347.13113	− 7.285
4	4.89	Vanillic acid	Organic acids	C_8_H_8_O_4_	[M − H]^−^	167.03498	167.03365	− 1.408
5	5.55	Forsythoside E	Phenylethanoid glycosides	C_20_H_30_O_12_	[M − H]^−^	461.16645	461.16504	− 0.678
5	5.56				[M + H]^+^	463.181	463.18076	− 0.524
6	5.79	Peiioside A	Organic acids	C_26_H_36_O_17_	[M − H]^−^	619.18797	619.18634	− 2.640
7	6.56	3,4-dihydroxybenzoic acid	Organic acids	C_7_H_6_O_4_	[M − H]^−^	153.01933	153.01788	− 2.321
8	6.56	Orthoanisic acid	Organic acids	C_8_H_8_O_3_	[M − H]^−^	151.04007	151.03867	− 9.252
9	6.73	Caffeic acid	Organic acids	C_9_H_8_O_4_	[M − H]^−^	179.03498	179.03362	− 1.481
10	6.78	Pinnatifidanoid A	Terpenoids	C_13_H_24_O_3_	[M + H]^+^	229.17982	229.17966	− 0.703
11	6.79	4-hydroxy cinnamic aicd	Organic acids	C_9_H_8_O_3_	[M − H]^−^	163.04007	163.03865	− 1.966
12	6.79	Methoxyquinol4-O-[(5-O-trans-p-caffeoyl)-β-D-apiofuranosyl-(1 → 2)-β-D-glucopyranoside]	Organic acids	C_27_H_32_O_15_	[M − H]^−^	595.16684	595.16559	− 0.157
13	6.84	Cistanoside F	Organic acids	C_21_H_28_O_13_	[M − H]^−^	487.14571	487.14453	− 2.431
14	7.06	Daidzin	Flavonoids	C_21_H_20_O_9_	[M − H]^−^	415.10346	415.10226	− 0.356
14	7.07				[M + H]^+^	417.11801	417.11786	− 0.356
15	7.16	Tuberonic acid glucoside	Organic acids	C_18_H_28_O_9_	[M − H]^−^	387.16606	387.16492	− 2.933
15	7.17				[M + H]^+^	389.18061	389.18011	− 1.282
16	7.49	Melilotoside	Organic acids	C_15_H_18_O_8_	[M − H]^−^	325.09289	325.09207	0.849
17	7.61	Vanillin	Phenols	C_8_H_8_O_3_	[M − H]^−^	151.04007	151.03867	− 1.990
17	7.66				[M + H]^+^	153.05462	153.05457	− 0.331
18	7.65	Syringic acid	Organic acids	C_9_H_10_O_5_	[M − H]^−^	197.04555	197.04427	− 0.913
19	7.78	β-OH-poliumoside	Phenylethanoid glycosides	C_35_H_46_O_20_	[M − H]^−^	785.25097	785.24927	− 0.764
20	7.80	Echinacoside	Phenylethanoid glycosides	C_35_H_46_O_20_	[M − H]^−^	785.25097	785.24927	− 0.764
21	8.41	Samioside	Phenylethanoid glycosides	C_34_H_44_O_19_	[M − H]^−^	755.2404	755.23816	− 1.516
22	8.69	5-methylisophthalic acid monomethyl ester	Organic acids	C_10_H_10_O_4_	[M − H]^−^	193.05063	193.04936	− 0.908
23	8.80	Poliumoside	Phenylethanoid glycosides	C_35_H_46_O_19_	[M − H]^−^	769.25605	769.25409	− 1.125
24	8.81	Luteolin 3′-O-glucuronide	Flavonoids	C_21_H_18_O_12_	[M + H]^+^	463.0871	463.08676	− 0.739
24	8.84				[M − H]^−^	461.07255	461.07138	− 0.157
25	8.86	Acteoside	Phenylethanoid glycosides	C_29_H_36_O_15_	[M − H]^−^	623.19814	623.19647	− 0.925
26	8.91	Forsythoside B	Phenylethanoid glycosides	C_34_H_44_O_19_	[M − H]^−^	755.2404	755.23822	− 1.437
27	9.10	Alyssonoside	Phenylethanoid glycosides	C_35_H_46_O_19_	[M − H]^−^	769.25605	769.25403	− 1.203
28	9.25	Isoacteoside	Phenylethanoid glycosides	C_29_H_36_O_15_	[M − H]^−^	623.19814	623.19641	− 1.022
29	9.26	Loliolide	Terpenoids	C_11_H_16_O_3_	[M + H]^+^	197.11722	197.1172	− 0.106
30	9.47	2′-acetyl forsythoside B	Phenylethanoid glycosides	C_36_H_46_O_20_	[M − H]^−^	797.25097	797.24915	− 0.903
31	9.49	Longissimoside B	Phenylethanoid glycosides	C_36_H_48_O_19_	[M − H]^−^	783.2717	783.2699	− 0.901
32	9.53	Chrisin-6,8-C-glycosides	Flavonoids	C_27_H_30_O_14_	[M + H]^+^	579.17083	579.17041	− 0.437
33	9.54	Dehydroxy forsythoside B	Phenylethanoid glycosides	C_34_H_44_O_18_	[M − H]^−^	739.24549	739.24396	− 0.583
34	9.61	Genistin	Flavonoids	C_21_H_20_O_10_	[M + H]^+^	433.11292	433.1127	− 0.515
35	9.66	Apigenin-7-glucuronide	Flavonoids	C_21_H_18_O_11_	[M + H]^+^	447.09219	447.09192	− 0.599
35					[M − H]^−^	445.07763	445.07648	− 0.130
36	9.77	Blumenol C	Terpenoids	C_13_H_22_O_2_	[M + H]^+^	211.16926	211.16899	− 1.262
37	9.84	Syringalide A 3′-α-l-rhamnopyranoside	Phenylethanoid glycosides	C_29_H_36_O_14_	[M − H]^−^	607.20323	607.20178	− 0.580
38	9.97	Cynaroside	Flavonoids	C_21_H_20_O_11_	[M − H]^−^	447.09328	447.09222	− 2.381
39	10.08	Chrysoeriol-7-O-β-D-glucopyranoside	Flavonoids	C_22_H_22_O_11_	[M − H]^−^	461.10893	461.23822	− 2.169
40	10.21	Ferulic acid	Organic acids	C_10_H_10_O_4_	[M − H]^−^	193.05063	193.04939	− 0.753
41	10.32	O-(methoxycarbonyl) phenylacetic acid	Organic acids	C_10_H_10_O_4_	[M − H]^−^	193.05063	193.04959	− 0.753
42	10.48	Philonotisflavone	Flavonoids	C_30_H_26_O_14_	[M − H]^−^	609.12498	609.20703	− 1.114
43	10.48	2′-acetylacteoside	Phenylethanoid glycosides	C_31_H_38_O_16_	[M − H]^−^	665.20871	665.20709	− 0.784
44	10.95	Daidzein	Flavonoids	C_15_H_10_O_4_	[M + H]^+^	255.06519	255.06493	− 1.001
44					[M − H]^−^	253.05063	253.04974	− 3.525
45	11.17	3,3′,4′,5,7-pentamethoxyflavone	Flavonoids	C_20_H_20_O_7_	[M − H]^−^	371.11363	371.1124	− 0.349
46	11.47	Luteolin	Flavonoids	C_15_H_10_O_6_	[M + H]^+^	287.05501	287.05487	− 0.503
46	11.48				[M − H]^−^	285.04046	285.03973	− 2.566
47	11.79	6-O-trans-cinnamoylphlorigidoside b	Terpenoids	C_29_H_36_O_13_	[M − H]^−^	591.20831	591.20691	− 0.520
48	11.85	Acacetin-7-O-β-glucuronide	Flavonoids	C_22_H_20_O_11_	[M − H]^−^	459.09328	459.09229	− 2.166
49	12.06	5,7,3′,4′-tetrahydroxy-3-methoxyflavanone	Flavonoids	C_16_H_12_O_7_	[M − H]^−^	315.05103	315.05032	− 2.241
49	12.06				[M + H]^+^	317.06558	317.06543	− 0.471
50	12.24	Benzyl-4′- hydroxy-benzoyl-3′-O-β-D-glucopyranoside	Organic acids	C_20_H_22_O_9_	[M − H]^−^	405.11911	405.11813	− 2.408
51	13.04	Abieta-8,11,13,15-tetraen-18-oic acid	Terpenoids	C_20_H_26_O_2_	[M + H]^+^	299.20056	299.20035	− 0.691
52	13.06	5,7,4′-trihydroxy-3′-methoxyflavanone	Flavonoids	C_16_H_12_O_6_	[M − H]^−^	299.05611	299.05569	− 1.410
52	13.07				[M + H]^+^	301.07066	301.07034	− 1.078
53	13.17	5,7,2′,6′-tetrahydroxyflavone	Flavonoids	C_15_H_10_O_6_	[M − H]^−^	285.04046	285.03967	− 2.776
53	13.18				[M + H]^+^	287.05501	287.05487	− 0.503
54	13.27	2α,3β,19α,23-tetrahydroxy-12-ene-28-oleanolic acid	Terpenoids	C_30_H_48_O_6_	[M − H]^−^	503.33781	503.3367	− 0.031
55	13.72	Cetraric acid	Organic acids	C_20_H_18_O_9_	[M − H]^−^	401.08781	401.08667	− 0.096
56	13.77	8α,9α,13α,14α-diepoxyabietan-18-oic acid	Terpenoids	C_20_H_30_O_4_	[M − H]^−^	333.20713	333.20627	− 2.589
57	14.04	7,8-epoxy-1(12)-caryophyllene-9β-ol	Terpenoids	C_15_H_24_O_2_	[M + H]^+^	237.18491	237.18475	− 0.660
58	14.46	Isorhamnetin	Flavonoids	C_16_H_12_O_7_	[M + H]^+^	317.06558	317.06543	− 0.471
58	14.49				[M − H]^−^	315.05103	315.15952	− 2.336
59	15.04	Rhamnazin	Flavonoids	C_17_H_14_O_7_	[M − H]^−^	329.06668	329.0657	− 2.966
60	15.62	2α,3β,22β,23-tetrahydroxyursolic-12-en-28-oic acid	Terpenoids	C_30_H_48_O_6_	[M − H]^−^	503.33781	503.33682	− 2.150
61	15.66	Callicarpone	Terpenoids	C_20_H_28_O_4_	[M − H]^−^	331.19148	331.19061	− 2.635
62	15.77	Accacetin	Flavonoids	C_16_H_12_O_5_	[M − H]^−^	283.0612	283.06033	− 3.062
63	17.02	CH_3_O-pectolinarigenin	Flavonoids	C_18_H_16_O_7_	[M − H]^−^	343.08233	343.0813	− 2.991
64	17.56	Euscaphic acid	Terpenoids	C_30_H_48_O_5_	[M − H]^−^	487.3429	487.34152	− 2.827
65	17.85	Apigenin	Flavonoids	C_15_H_10_O_5_	[M − H]^−^	269.04555	269.0448	− 2.775
66	18.43	7β-hydroxy dehydroabietic acid	Terpenoids	C_20_H_28_O_3_	[M − H]^−^	315.19657	315.19583	− 2.341
67	19.12	Pentandralactone	Terpenoids	C_20_H_28_O_4_	[M − H]^−^	331.19148	331.19067	− 2.454
68	19.62	16,17-dihydroxy-3-oxophyllocladane	Terpenoids	C_20_H_30_O_3_	[M − H]^−^	317.21222	317.21118	− 3.178

**Table 2:** Identification of 31 migrating constituents absorbed into serum by UHPLC-Q Exactive Orbitrap MS.

No	T_R_	Identification	Category	Formula	Ion mode	Theo. mass (m/z)	Obser. mass (m/z)	Error (ppm)
1	4.40	3,4,5-trimethoxyphenyl-β-D-glucopyranoside	Phenols	C_15_H_22_O_9_	[M + H]^+^	347.13366	347.1315	− 2.159
2	5.51	Forsythoside E	Phenylethanoid glycosides	C_20_H_30_O_12_	[M − H]^−^	461.16645	461.16504	− 1.409
3	5.78	Peiioside A	Organic acids	C_26_H_36_O_17_	[M − H]^−^	619.18797	619.18524	− 2.733
4	6.56	3,4-dihydroxybenzoic acid	Organic acids	C_7_H_6_O_4_	[M − H]^−^	153.01933	153.01791	− 1.422
5	6.73	Caffeic acid	Organic acids	C_9_H_8_O_4_	[M − H]^−^	179.03498	179.0336	− 1.382
6	6.79	4-hydroxy cinnamic aicd	Organic acids	C_9_H_8_O_3_	[M − H]^−^	163.04007	163.0387	− 1.367
7	7.18	Tuberonic acid glucoside	Organic acids	C_18_H_28_O_9_	[M − H]^−^	387.16606	387.16498	− 1.076
8	7.45	Melilotoside	Organic acids	C_15_H_18_O_8_	[M − H]^−^	325.09289	325.09195	− 0.941
9	7.80	Echinacoside	Phenylethanoid glycosides	C_35_H_46_O_20_	[M − H]^−^	785.25097	785.25122	0.253
10	7.82	β-OH-poliumoside	Phenylethanoid glycosides	C_35_H_46_O_20_	[M − H]^−^	785.25097	785.24884	− 2.127
11	8.45	Samioside	Phenylethanoid glycosides	C_34_H_44_O_19_	[M − H]^−^	755.2404	755.23877	− 1.632
12	8.66	5-methylisophthalic acid monomethyl ester	Organic acids	C_10_H_10_O_4_	[M − H]^−^	193.05063	193.04933	− 1.302
13	8.83	Acteoside	Phenylethanoid glycosides	C_29_H_36_O_15_	[M − H]^−^	623.19814	623.19666	− 1.483
14	8.87	Poliumoside	Phenylethanoid glycosides	C_35_H_46_O_19_	[M − H]^−^	769.25605	769.25482	− 1.232
15	8.89	Forsythoside B	Phenylethanoid glycosides	C_34_H_44_O_19_	[M − H]^−^	755.2404	755.23834	− 2.062
16	9.08	Alyssonoside	Phenylethanoid glycosides	C_35_H_46_O_19_	[M − H]^−^	769.25605	769.25482	− 1.232
17	9.21	Isoacteoside	Phenylethanoid glycosides	C_29_H_36_O_15_	[M − H]^−^	623.19814	623.19635	− 1.793
18	9.49	Longissimoside B	Phenylethanoid glycosides	C_36_H_48_O_19_	[M − H]^−^	783.2717	783.26971	− 1.992
19	9.66	Apigenin-7-glucuronide	Flavonoids	C_21_H_18_O_11_	[M − H]^−^	445.07763	445.07599	− 1.645
19	9.66	Apigenin-7-glucuronide	Flavonoids	C_21_H_18_O_11_	[M + H]^+^	447.09219	447.09158	− 0.608
20	10.08	Chrysoeriol-7-O-β-D-glucopyranoside	Flavonoids	C_22_H_22_O_11_	[M − H]^−^	461.10893	461.10547	− 3.465
21	10.21	Ferulic acid	Organic acids	C_10_H_10_O_4_	[M − H]^−^	193.05063	193.04959	− 1.042
22	10.21	O-(methoxycarbonyl) phenylacetic acid	Organic acids	C_10_H_10_O_4_	[M − H]^−^	193.05063	193.04959	− 1.042
23	10.93	Daidzein	Flavonoids	C_15_H_10_O_4_	[M − H]^−^	253.05063	253.05014	− 0.492
23	10.93	Daidzein	Flavonoids	C_15_H_10_O_4_	[M + H]^+^	255.06519	255.065	− 0.185
24	11.24	3,3′,4′,5,7-pentamethoxyflavone	Flavonoids	C_20_H_20_O_7_	[M − H]^−^	371.11363	371.11301	0.481
25	13.02	5,7,4′-trihydroxy-3′-methoxyflavanone	Flavonoids	C_16_H_12_O_6_	[M − H]^−^	299.05611	299.05569	− 0.421
26	13.27	2α,3β,19α,23-tetrahydroxy-12-ene-28-oleanolic acid	Terpenoids	C_30_H_48_O_6_	[M − H]^−^	503.33781	503.33737	− 0.442
27	15.59	2α,3β,22β,23-tetrahydroxyursolic-12-en-28-oic acid	Terpenoids	C_30_H_48_O_6_	[M − H]^−^	503.33781	503.33737	− 0.442
28	15.61	Callicarpone	Terpenoids	C_20_H_28_O_4_	[M − H]^−^	331.19148	331.19104	− 0.443
29	17.78	Apigenin	Flavonoids	C_15_H_10_O_5_	[M − H]^−^	269.04555	269.0451	− 0.447
30	19.12	Pentandralactone	Terpenoids	C_20_H_28_O_4_	[M − H]^−^	331.19148	331.19104	− 0.443
31	19.64	16,17-dihydroxy-3-oxophyllocladane	Terpenoids	C_20_H_30_O_3_	[M − H]^−^	317.21222	317.21118	− 1.038

### CK-containing serum decreased iNOS but not COX-2 protein levels in LPS-stimulated RAW264.7 cells

**Figure 8:**
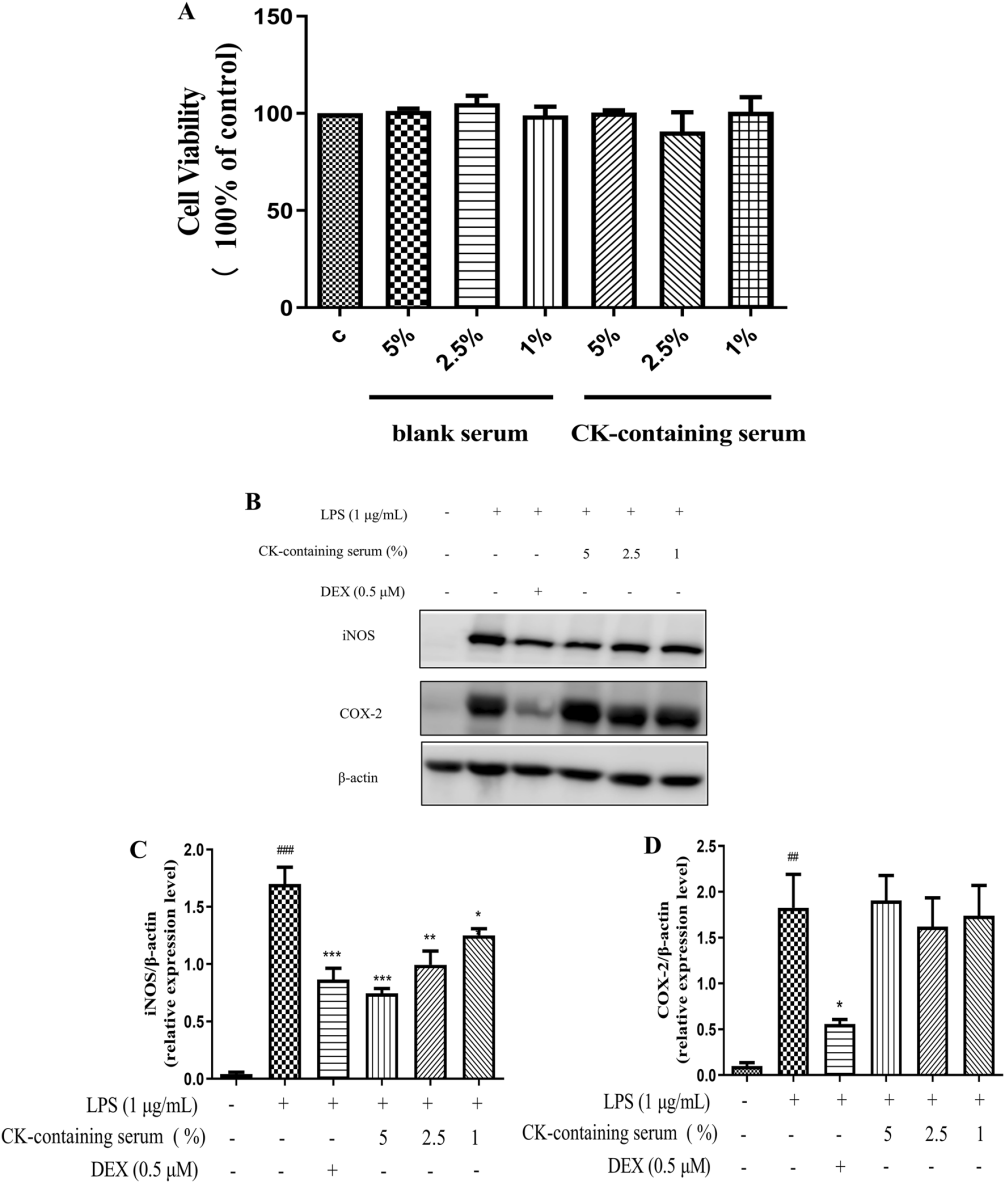
CK-containing serum decreased iNOS but not COX-2 protein levels in LPS-stimulated RAW264.7 cells. (**A**) Effect of CK-containing serum and blank serum on the viability of RAW264.7 cells. (**B**–**D**) Effect of CK-containing serum on the protein expressions of COX-2 and iNOS in LPS-stimulated RAW264.7 cells. Cells were pretreated with CK-containing serum (1%, 2.5% and 5%) or DEX for 1 h, and then stimulated with LPS (1 µg/mL) for 18 h. The protein expression of iNOS and COX-2 was analysed by western blotting. The data are presented as the means ± SDs of three experiments. ^##^*P* < 0.01, ^###^*P* < 0.001, versus the control group. ^*^P < 0.05, ^**^P < 0.01, ^***^P < 0.001 versus the LPS-stimulated group.

Adding 5% medicated serum did not change the viability of macrophages. Based on the results of the MTT assay, 1%, 2.5% and 5% CK were used for the following experiments (Fig. 8A). When RAW264.7 cells were cultured with LPS for 18 h, iNOS and COX-2 expression was strongly upregulated. Compared with the those in LPS group, the protein level of iNOS and COX-2 in the DEX group significantly decreased. The levels of iNOS induced by CK-containing serum at concentrations of 1%, 2.5% and 5% were significantly reduced (Fig. 8B–C). However, CK-containing serum did not markedly decrease COX-2 protein expression (Fig. 8D), which was consistent with the previous results.

### CK-containing serum reduced NO, TNF-α, and IL-6 production in LPS- stimulated RAW264.7 cells

**Figure 9:**
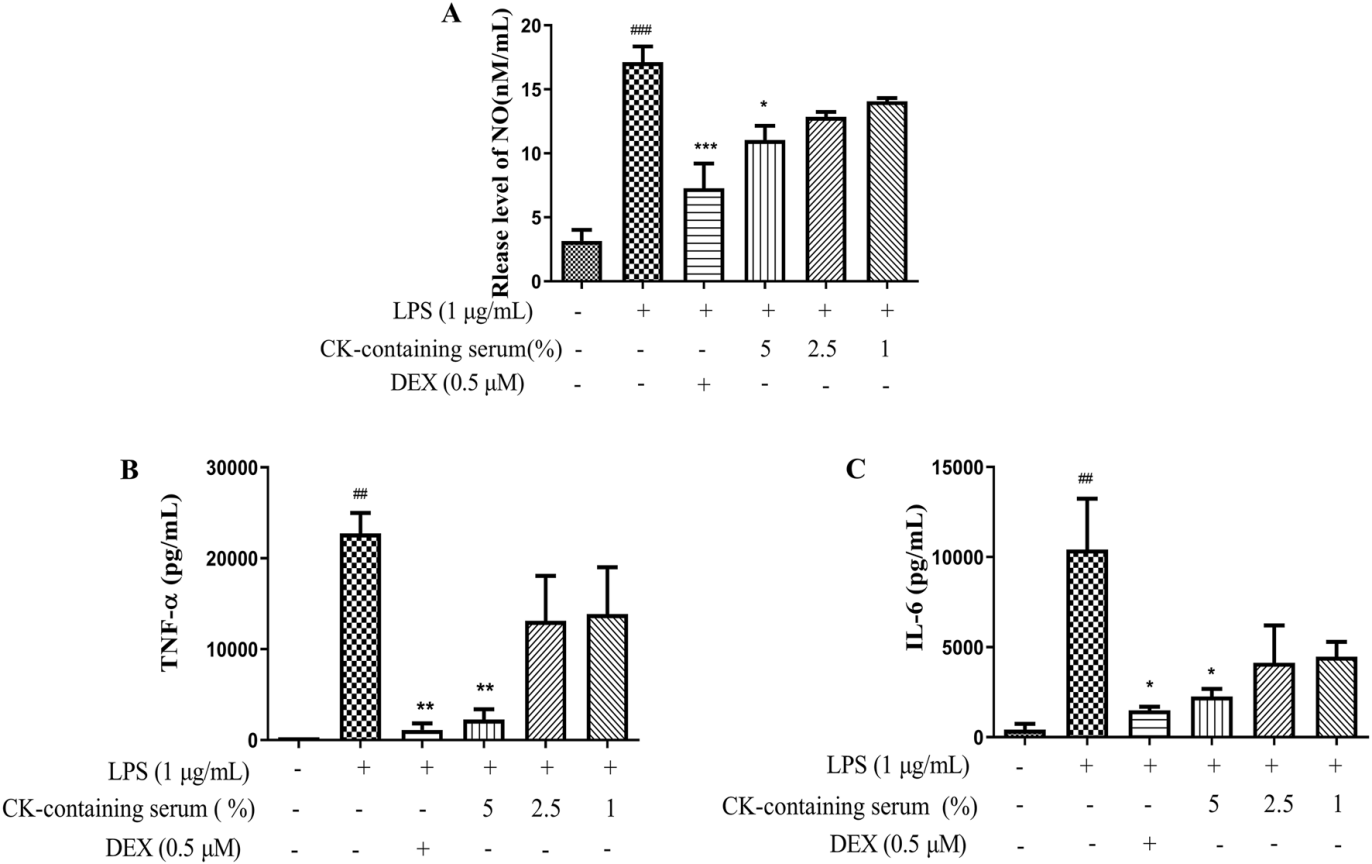
CK-containing serum reduced the production of NO and inflammatory cytokines in LPS-stimulated RAW264.7 cells. The release of NO into the culture supernatant was examined with a Griess reagent kit (**A**). The levels of TNF-α (**B**) and IL-6 (**C**) in the culture medium were determined using ELISA kits. The values are expressed as the means ± SDs (n = 3). Different superscript letters indicate statistical significance. ^##^*P* < 0.01, ^###^*P* < 0.001, versus the control group. ^*^P < 0.05, ^**^P < 0.01, ^***^P < 0.001 versus the LPS-stimulated group.

After LPS induction, NO, TNF-α, and IL-6 levels in RAW264.7 cells were significantly increased. Compared with the those in LPS group, the NO, IL-6, and TNF-α production in the DEX group significantly decreased. CK-containing serum reduced the release of NO in the high-concentration group and decreased the levels of these inflammatory cytokines (Fig. 9). Taken together, these data indicated that the CK-containing serum inhibited LPS-induced inflammatory damage in RAW264.7 cells.

## Discussion

Inflammation is an adaptive response that restores tissue function and homeostasis^33^. However, this response generally occurs at the expense of many other physiological processes, indicating that excessive and chronic inflammatory responses cause further damage to the human body^34^. Many studies have indicated that regulating inflammatory responses can treat various diseases and forestall disease complications. However, the use of common anti-inflammatory drugs in the clinic is restricted by side effects. CK is a traditional Chinese medicine, that is thought to have anti-inflammatory properties. However, to our knowledge, no published studies have evaluated its anti-inflammatory effects in vivo or in vitro. This study investigated the anti-inflammatory effects of CK water extract by in vivo and in vitro for the first time.

The anti-inflammatory effects of many traditional Chinese medicine extracts have been confirmed in a classic LPS-induced inflammatory cell model^35^. Therefore, we selected LPS-stimulated RAW264.7 cells as an inflammatory model for in vitro experiment. The steroidal drug dexamethasone (DEX) is frequently used in the clinical treatment of various inflammatory diseases^36,37^. Therefore, we selected DEX as a positive control to evaluate the anti-inflammatory activity of CK.

**Figure 10:**
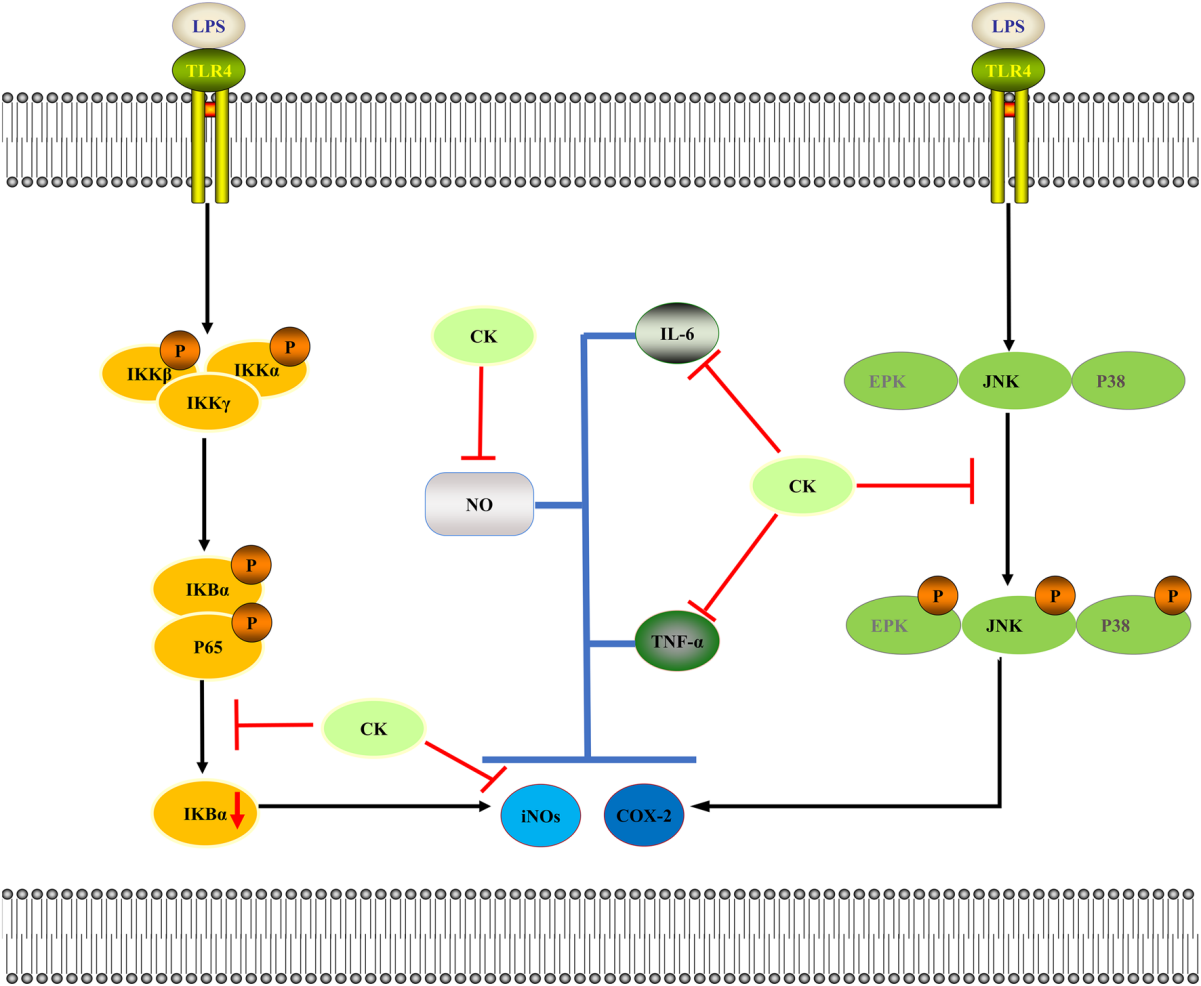
Proposed molecular mechanism by which CK inhibits LPS-stimulated macrophages. LPS, which is a common macrophage activator, can bind to TLR4, thereby activating macrophages through the NF-κB and MAPK signaling pathway. Activation of the NF-κB signaling pathway leads to the decrease of IκBα expression, which increase the levels of proinflammatory mediators (iNOS) and cytokines (TNF-α and IL-6). Activation of the MAPK signaling pathway leads to the activation of JNK, p38, and ERK, which increase the levels of proinflammatory mediators (iNOS) and cytokines (TNF-α and IL-6). CK can inhibit the decrease of IκBα expression and the activation of JNK, p38, and ERK, thereby decreasing the levels of proinflammatory mediators (iNOS) and cytokines (TNF-α and IL-6). In addition, CK-mediated inhibition of the expression of proinflammatory mediators (iNOS) and cytokines (TNF-α and IL-6) can ultimately suppress the release of NO.

The anti-inflammatory effect of plant extracts involves inhibiting the secretion of proinflammatory factors and reducing protein expression by affecting related signaling pathways, but not stimulating cells through toxic effects. Therefore, we performed an MTT experiment. iNOS and COX-2 are regulatory enzymes associated with NO release and PGE_2_ production, respectively. Many studies have investigated the role of NO in acute and chronic inflammation. NO is also considered to be a mediator of rheumatoid arthritis^38^, and a previous study suggested that NO was involved in RA pathogenesis by boosting blood flow in the synovium and regulating synovial function and articular chondrocytes. PGEs, which are active substances containing unsaturated fatty acids, exist widely in animals and humans and include PGE_2_ and thromboxane A_2_ (TXA_2_)^39^. Previous studies have shown that PGE_2_ and TXA_2_ are regulated by COX-2 protein expression. Therefore, suppressing the levels of NO and PGE_2_ is important for the development of anti-inflammatory medicines. Our results showed that 0–100 µg/mL CK water extract had no effect on the viability of nonactivated cells or LPS-induced activated cells. The CK water extract could inhibit NO release by suppressing iNOS expression, and this inhibitory effect was stronger than that of DEX. However, the CK water extract had no significant effect on the expression of COX-2, suggesting that CK might have a lower cardiovascular risk than DEX (Fig. 10).

In inflammatory responses, activated macrophages release large amounts of cytokines, further increasing inflammatory responses^40^. NO, TNF-α and IL-6 are produced in acute and chronic inflammatory diseases^41^. In this study, all of these inflammatory cytokines were increased by LPS stimulation and were reduced by CK (Fig. 10). These results showed that CK had strong anti-inflammatory activity. Thus, further investigation of its anti-inflammatory potential is necessary.

Typically, inflammation is controlled through the NF-κB and MAPK signaling pathways^42^. After cells are stimulated by LPS, NF-κB is released and quickly transferred to the nucleus, where it activates the transcription of its target genes^43^. MAPKs can regulate cytokine, iNOS and COX-2 gene expression^44^, and are activated by LPS-stimulation. Three parallel MAPK pathways (JNK, ERK and P38) are activated in mammalian cells^45^. Our results showed that CK strongly inhibited JNK, ERK and P38 phosphorylation in the MAPK pathway, and increased IκBα expression in the NF-κB signaling pathway. However, CK did not inhibit p65 phosphorylation in NF-κB signaling pathway. Based on these findings, it can be inferred that CK exerts an anti-inflammatory effect by suppressing activation of the MAPK and NF-κB signaling pathway (Fig. 10).

Moreover, for the first time, we assessed the anti-inflammatory effects of CK by experiment in vivo. The λ-carrageenan induced rat foot swelling model is a classic acute inflammatory model used for new drug development. The CIA rat model is a standard model used in preclinical studies^46^. Therefore, in this study, both of above these animal models were used to assess the therapeutic effects of CK on acute and chronic inflammation. Our results indicated that λ-carrageenan alleviated foot swelling in rat model of foot swelling and reduced the arthritis score and incidence of arthritis in a CIA rat model. These findings are consistent with the in vitro results described above, indicating that CK exerts therapeutic effects on a rat model and may have be used to treat acute and chronic inflammatory conditions, especially rheumatoid arthritis. In brief, CK has been shown to exert strong anti-inflammatory effects on acute and chronic inflammation, and as a new anti-inflammatory traditional Chinese medicine, it has the potential to be further investigated and developed.

The effective constituents of drugs are the foundation for therapeutic action^47^. Traditional Chinese medicine has many components, but only some compounds that migrate in serum are active components^48,49^. Although, the organic acids, phenylethanoid glycosides, and flavonoids of CK have been studied by several research groups^32^, this study examined the components of CK found in rat serum for the first time. Our results showed that a total of 31 components of CK were found in rat serum. We hypothesized that the anti-inflammatory effects of CK were attributable to these factors.

Serum pharmacology involves pharmacological observation of herbal drug-containing serum. We examined the anti-inflammatory effects of CK-containing serum for the first time. Our results showed that blank serum and serum containing 5–1% CK had no effect on cell viability. Consistent with the effect of CK water extract, CK-containing serum regulated iNOS but not COX-2 protein expression. CK-containing serum also regulated the release of NO, indicating that the anti-inflammatory effects of CK water extract were attributable to these components. Furthermore, we examined the effect of CK-containing serum on proinflammatory cytokines. The results showed that CK-containing serum reduced NO, TNF-α and IL-6 levels, which was consistent with the effect of CK water extract. These data further verified that the components of CK within serum had major anti-inflammatory effects. However, there are still limitations to this study. We have not been able to further identify all the anti-inflammatory components in CK, or effectively predict the targets of blood components in diseases of concern. We examined the key targets of the anti-inflammatory effects of CK through pathway analysis, functional analysis, interaction network analysis and other methods. In the future, further investigation and screening of anti-inflammatory components that enter the blood is necessary.

## Conclusions

Overall, the anti-inflammatory effect of CK was investigated in vivo and in vitro for the first time. Our results showed that CK exerted notable anti-inflammatory effects and exhibited unique advantages, including (1) inhibiting NO production, leading to anti-inflammatory effects; (2) downregulating the expression of inflammatory cytokines to inhibit the progression of the inflammatory response; (3) suppressing iNOS protein expression but not COX-2 protein expression to avoid cardiovascular risk; (4) inhibiting the activation of the MAPK and NF-κB signaling pathway to exert anti-inflammatory effects; and (5) reducing the swelling rate, arthritis score and incidence to exert therapeutic effects on acute and chronic inflammation. Qualitative analysis of CK-containing serum showed that 31 components of CK were found in rat serum. It is worth noting that CK-containing serum exhibited excellent anti-inflammatory activity, which is consistent with CK that of water extract. These results show that CK is a promising candidate treatment for inflammatory diseases, especially those associated with the MAPK pathway. Although more experiments are needed to systematically validate the anti-inflammatory activity of CK, this study helps to provides evidence for the use of CK in inflammatory disease treatments and further studies.

## Materials and methods

### Materials

*Callicarpa kwangtungensis* Chun water extract (Batch number: 220301, inspection batch number: YL2203003, forsythia glycoside B: 69.3 mg/g) was offered by Huaihua Zhenghao Pharmaceutical Co., Ltd (Extraction method according to Chinese Pharmacopoeia). Dulbecco’s Modifified Eagle’s medium (DMEM, C11995500BT) and Fetal Bovine Serum (FBS, 10,091–148) were all purchased from Themo Fisher Scientific, USA. Loading BUffer 5 × (BL502B) was obtained from Biosharp Life Science. Dexamethasone (DEX, D1756), Lipopolysacccharides (LPS, L2880-100MG), λ-carrageenan (Lot # BCBP8978V) were all obtained from Sigma, USA. Anti-mouse IgG (7076S), Anti-rabbit IgG (7074P2), Mouse anti-β-actin polyclonal antibody (3700S), iNOS (2982S), COX-2 (1282S) Rabbit antibody, p-38 (8690T), P-p38 (4511T), ERK (4695T), P-ERK (4370T), JNK (9252T), P-JNK (4668T), p-65 (8242T), P-p65 (3033T), and IκBα (9242S) were all obtained from Cell Signaling Technology, USA. Mouse IL-6 and TNF-α Enzyme-linked immunosorbent assay (A20620225) was obtained from ExCell Biotechnology Co., Ltd., Shanghai, China. Griess assay kit (S0021M) and Protein Quantification Kit (P0010S) were obtained from Beyotime Biotechnology. ECLIPSE PLUS C18 (100 × 2.1 mm, 1.8 µm) was obtained from Agilent. Bovine type II collagen (150517) and IFA (210076) were purchased from Chondrex. Methanol, formic acid, water, and acetonitrile in PLC grade (Fisher scientific) were procured.

### Preparation of CK water extracts

The impurities were removed, and the samples (dry stems and leaves) were moistened thoroughly, cut into small segments or tablets, and dried (60–70 °C), resulting in an 86–98% yield. The sample was mixed at a 1:3 ratio of medicinal materials to water, and heated at 60–80 °C. One sample was heated for 3 h and the other was heated 2 h, after which the decoction was filtered, and the two decoctions were combined. The filtrate was stored in the decoction tank, and the filtrate was concentrated into an extract with a relative density of approximately 1.28–1.32 (55–60 °C). The sample was placed into the material tray of the microwave vacuum dryer and dried into powder 75–80 °C.

### Cell culture

RAW264.7 cells (American Type Culture Collection) were incubated in complete medium consisting of DMEM, 10% FBS and 1% PS in an atmosphere containing 5% CO_2_ at 37 °C. After being dissolved by DMSO, the CK water extract was filtered through a 0.22 µm filter membrane, and further diluted with DMEM.

### Cell viability assay

RAW264.7 cells (1 × 10^5^ cells/mL) were evenly seeded and treated with different concentrations of CK water extract/serum for 24 h. LPS (1 µg/mL) was added one hour later. After 18 h, 10 µL of MTT (Bio Basic Inc) solution (1 mL of medium containing 5 mg of MTT) was added to each well and incubated for 4 h. The crystals were carefully dissolved in DMSO (100 µL/well), and examined using a microplate reader (BMG LABTECH, Germany).

### Nitric oxide (NO) assay

The experimental cells (2 × 10^5^ cells/mL) were cultured for 24 h and pretreated with CK water extract/ CK-containing serum or DEX for 1 h. Then, the culture solution was stimulated with LPS for 18 h. Next, the supernatant was collected and examined by a nitrite assay kit.

### Enzyme-linked immunosorbent assay (ELISA)

ELISA was used to measure IL-6 and TNF-α levels in LPS-induced RAW264.7 cells with or without pretreatment with CK water extract/CK-containing serum or DEX. The supernatants were collected and measured at 450 nm.

### Western blot assay

Cells (2 × 10^5^ cells/mL) were seeded onto a 12-well plate and incubated for 24 h and pretreated with CK water extract/ CK-containing serum or DEX for 1 h. Then the cells were stimulated with1 µg/mL LPS for 0.25 h (for MAPK- and NF-κB-associated proteins) or 18 h (for iNOS and COX-2). After the total proteins were separated by 10% SDS-PAGE, the proteins were transferred to a PVDF membrane. The membranes were incubated by the specific antibodies at 4 °C overnight and the secondary antibodies for 1 h. A laser imaging system was applicated for protein quantification.

### Ethics statement

All animal care and experiments were approved by the Ethics Committee of the National Research Centre and the Ethics Committee of Hunan University of Medicine [approval number: 2022(A01017)]. The study was carried out in accordance with the ARRIVE guidelines and all methods were performed in accordance with the relevant guidelines and regulations. These animals were euthanized according to the CO_2_ inhalation method in the AVMA Guidelines for the Euthanasia of Animals: 2020 Edition (equipment: CO_2_ euthanasia device Carbon dioxide euthanasia box/HOPE-MED8160, concentrations: 30% of the chamber or cage volume/min).

### Rat foot swelling model

Healthy and clean male SD rats (6–8 weeks old) were obtained from Hunan Slack Jingda Experimental Animal Co., Ltd., and divided into model group, CK (50 or 100 mg/kg) group and indomethacin (10 mg/kg) group. The rats were intragastrically administered CK or indomethacin. After 2 h, the rats were injected with 0.1 mL of 1% (w/v) λ-carrageenan to induce an acute inflammation model. A rat foot swelling tester (PV-200; Chengdu Taimeng Technology Co., Ltd.) was used to measure foot swelling (1 h, 2 h, 3 h, and 4 h) after λ-carrageenan-induced inflammation.

The swelling percentage was calculated according to the following formula: swelling rate (%) = (volume of toe after inflammation-volume of toe before inflammation)/volume of toe before inflammation × 100%.

### Collagen-induced arthritis (CIA) rat model

Male SD rats (220 ± 20 g average weight) were stochastically grouped into the model group, control group, CK (50 mg/kg) group and CK (100 mg/kg) group. Bovine type II collagen solution was emulsified with a mortar in an equal volume of incomplete Freund’s adjuvant.

The mixture was injected into the rats via the tail vein on ice. Seven days after the first injection of the emulsion, IFA was injected again to enhance immunity as described previously. The first foot measurement was performed at the time of the second immunization, and foot measurements were performed every other day after the second immunization.

The degree of arthritis was scored by the joint scoring method to obtain the arthritis index (the sum of the scores of each animal’s limbs), which was based on the degree and scope of joint swelling and deformation. The weight and organ indices of the rats were recorded.

### Preparation of serum samples

Twelve SD rats (220 ± 20 g) were stochastically divided into two groups (the control and CK groups). In the CK group, the rats were perfused with CK (1000 mg/kg) three times daily. After the seventh administration, the serum was collected, inactivated at 56 °C in a water bath for 15 min, and stored at − 80 °C. Methyl alcohol (four times the volume) was added, and the mixture was vortexed for 2 min. The supernatant was collected after centrifugation at 4 °C and 13,000 rpm for 10 min and then was dried with N2 at 40 °C. Then, methanol (150 µL) was added. The solution was mixed in an ultrasonic bath for 15 min. After centrifugation for 15 min at 13,000 rpm and being filtered through a 0.22 µm pore membrane, the samples were analyzed by LC–MS.

### LC–MS analysis

Qualitative analysis was performed with an ECLIPSE PLUS C18 (Agilent, 100 × 2.1 mm, 1.8 µm) at 40 °C on a UHPLC- Q Exactive Orbitrap MS (Thermo Electron, Bremen, Germany). The mobile phases consisted of the A phase (0.1% formic acid) and B phase (acetonitrile) and were run at a velocity of 0.3 mL/min with the following gradient: 0–1 min, 2% B; 2 min, 5% B; 5 min, 15% B; 15 min, 50% B; 20 min, 80% B; 24 min, 98% B; and 25 min, 2% B. A full scan (m/z: 120–1000) was performed by an Orbitrap analyser. Nitrogen (normalized collision energy: 30%, 40% and 60%) was used to generate the fragment ions, and PRM mode was used to obtain the MS2 data.

### Statistical analysis

The data were statistically analysed with GraphPad Prism 8.0 software. Differences were determined by Student’s t-test after one-way ANOVA and the log-rank (Mantel-Cox) test were used (*P* < 0.05).

### Ethics approval and consent to participate

All animal care and experiments were approved by the Ethics Committee of the National Research Centre and the Ethics Committee of Hunan University of Medicine [approval number: 2022(A01017)].

### Supplementary Information

Supplementary Information 1.

Supplementary Information 2.

Supplementary Information 3.

Supplementary Information 4.

Supplementary Information 5.

Supplementary Information 6.

Supplementary Information 7.

Supplementary Information 8.

Supplementary Information 9.

Supplementary Information 10.

Supplementary Information 11.

Supplementary Information 12.

Supplementary Information 13.

Supplementary Information 14.

Supplementary Information 15.

Supplementary Information 16.

Supplementary Information 17.

Supplementary Information 18.

Supplementary Information 19.

## Data Availability

All data generated or analyzed during this study are included in this published article.

## Abbreviations

CK: Callicarpa kwangtungensis Chun
NF-κB: Nuclear factor kappa-B
MAPK: Mitogen-activated protein kinase
COX-2: Cyclooxygenase-2
IL-6: Interleukin-6
NO: Nitric oxide
TNF-α: Tumor necrosis factor α
iNOS: Inducible nitric oxide synthase
DEX: Dexamethasone
RA: Rheumatoid arthritis
LPS: Lipopolysaccharide
MTT: Thiazolyl blue tetrazolium bromide
CIA: Collagen-induced arthritis
ELISA: Enzyme-linked immunosorbent assay
