# Supplementary figures and images for: Anti-inflammatory properties and characterization of water extracts obtained from Callicarpa kwangtungensis Chun using in vitro and in vivo rat models
Source: Sci Rep. 2024 May 14;14:11047. doi: 10.1038/s41598-024-61892-9 (PMC11094131; doi:10.1038/s41598-024-61892-9)

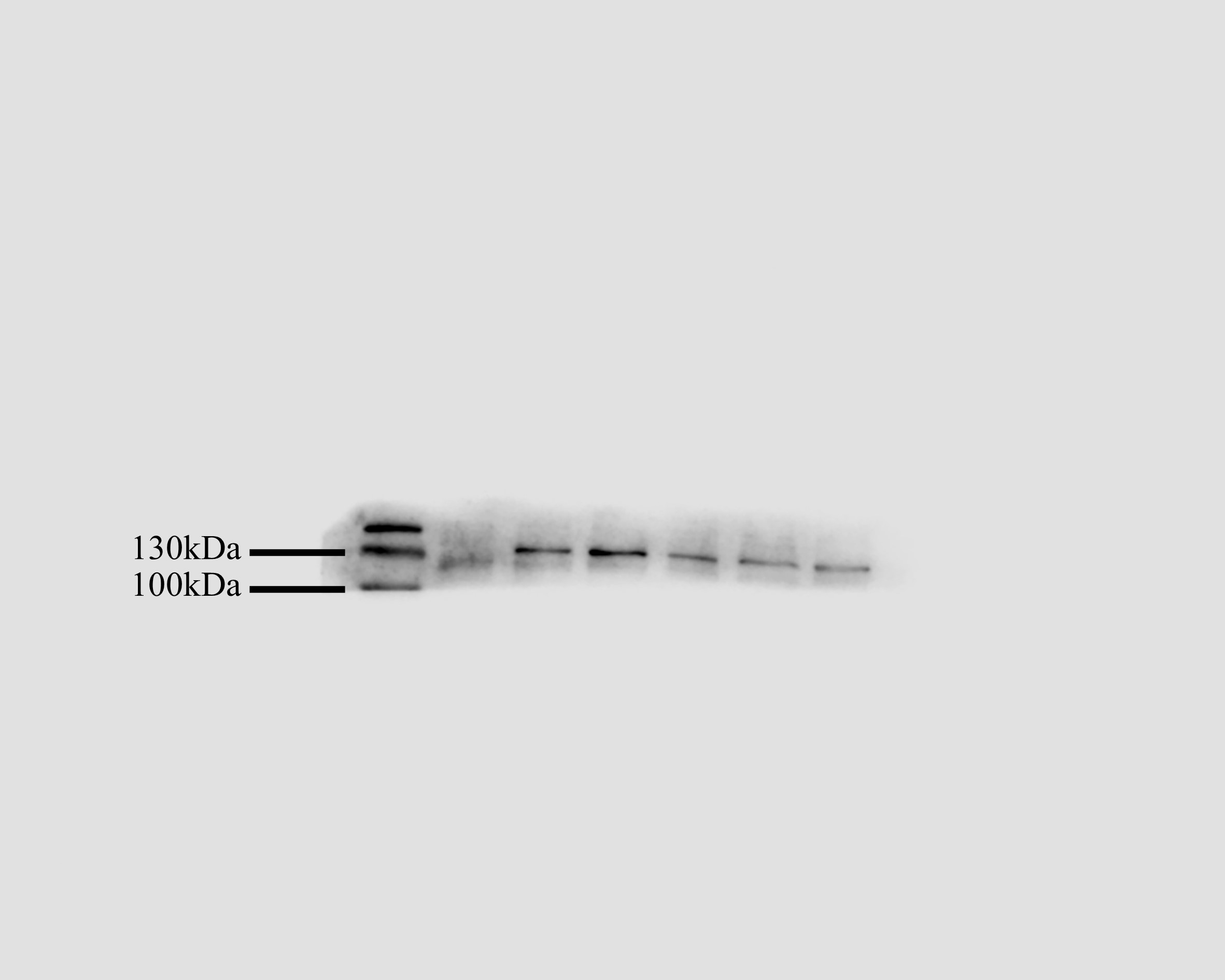

Supplement: Supplementary file 1 — Supplementary Information 1. [file 41598_2024_61892_MOESM1_ESM.jpg]

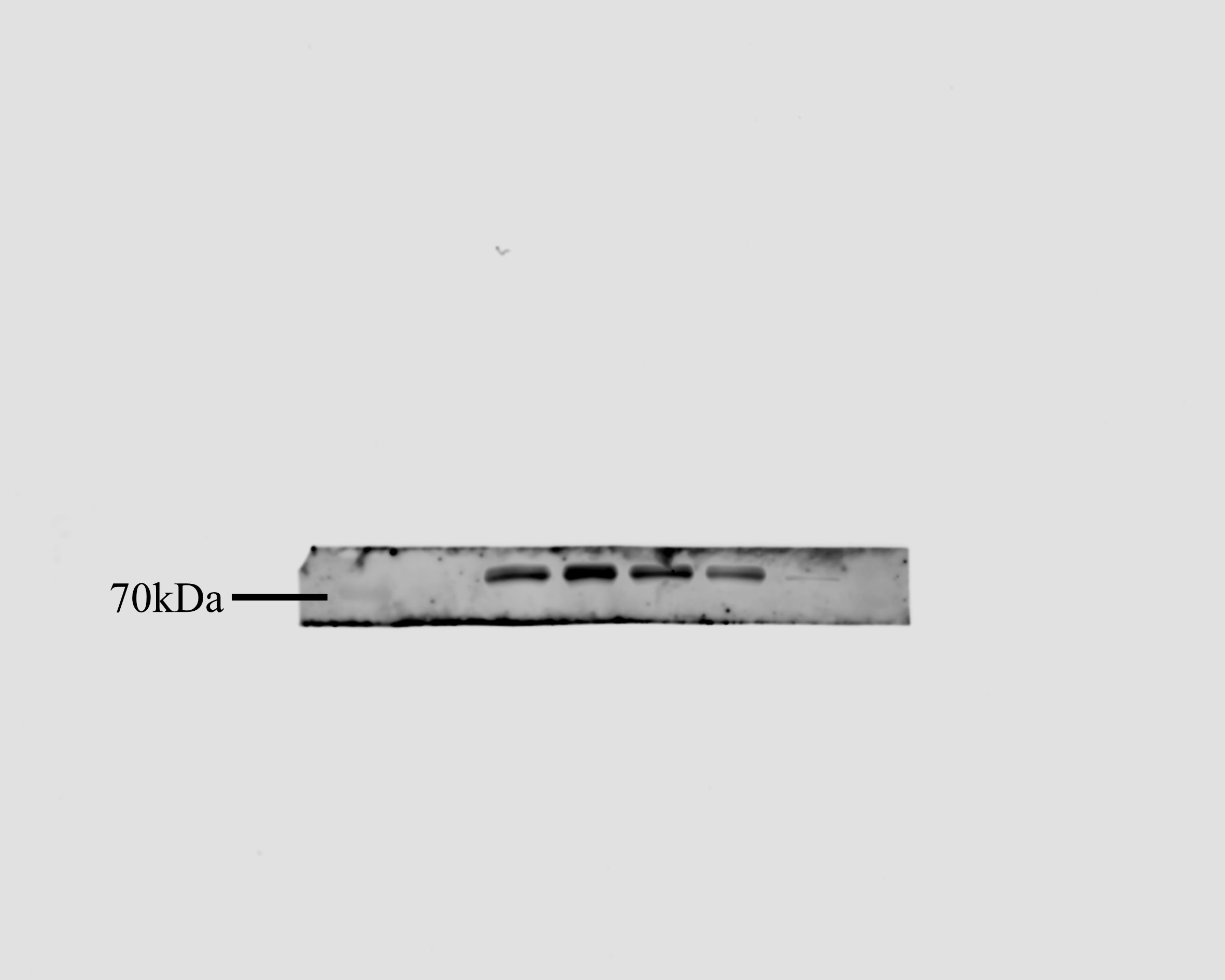

Supplement: Supplementary file 2 — Supplementary Information 2. [file 41598_2024_61892_MOESM2_ESM.jpg]

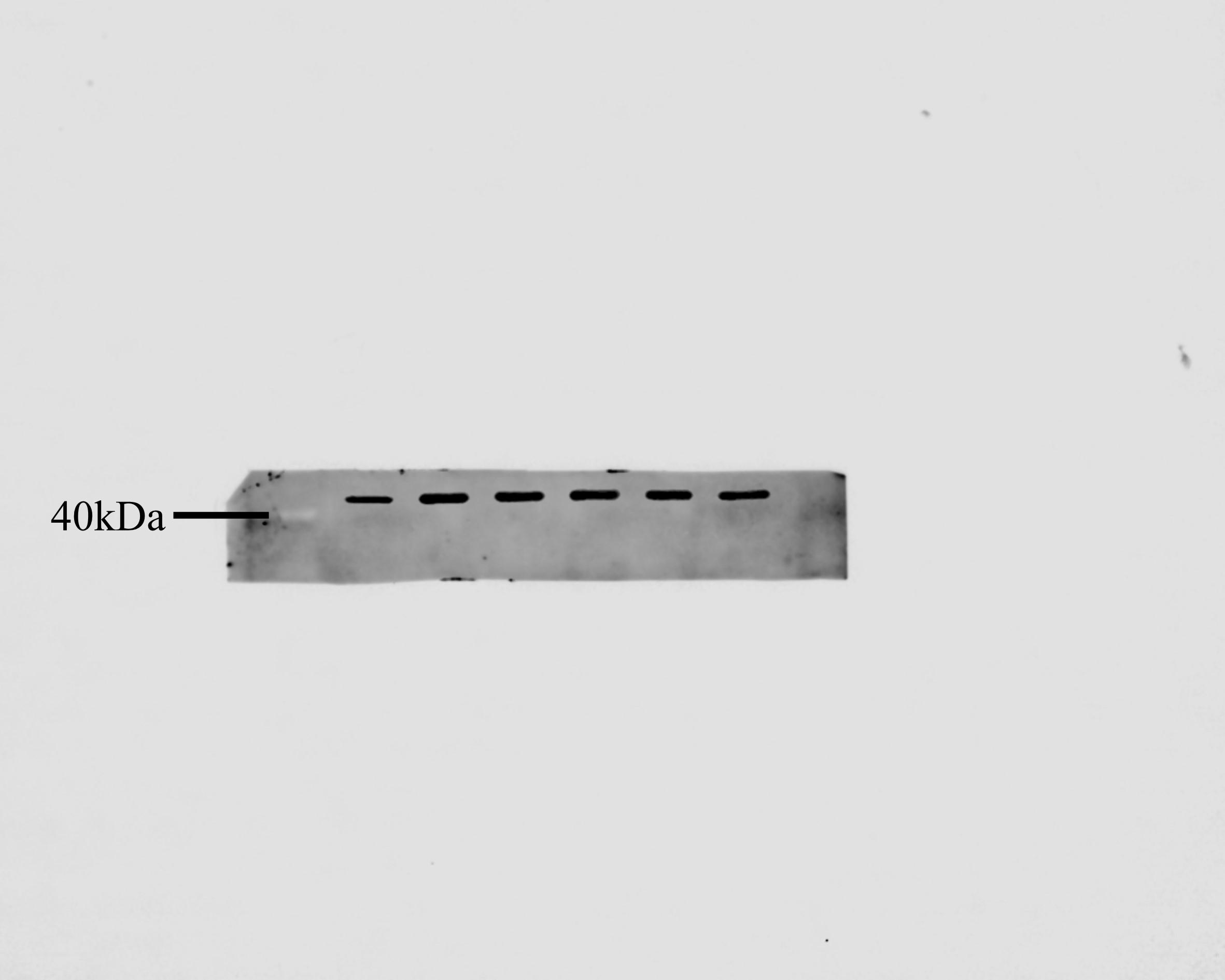

Supplement: Supplementary file 3 — Supplementary Information 3. [file 41598_2024_61892_MOESM3_ESM.jpg]

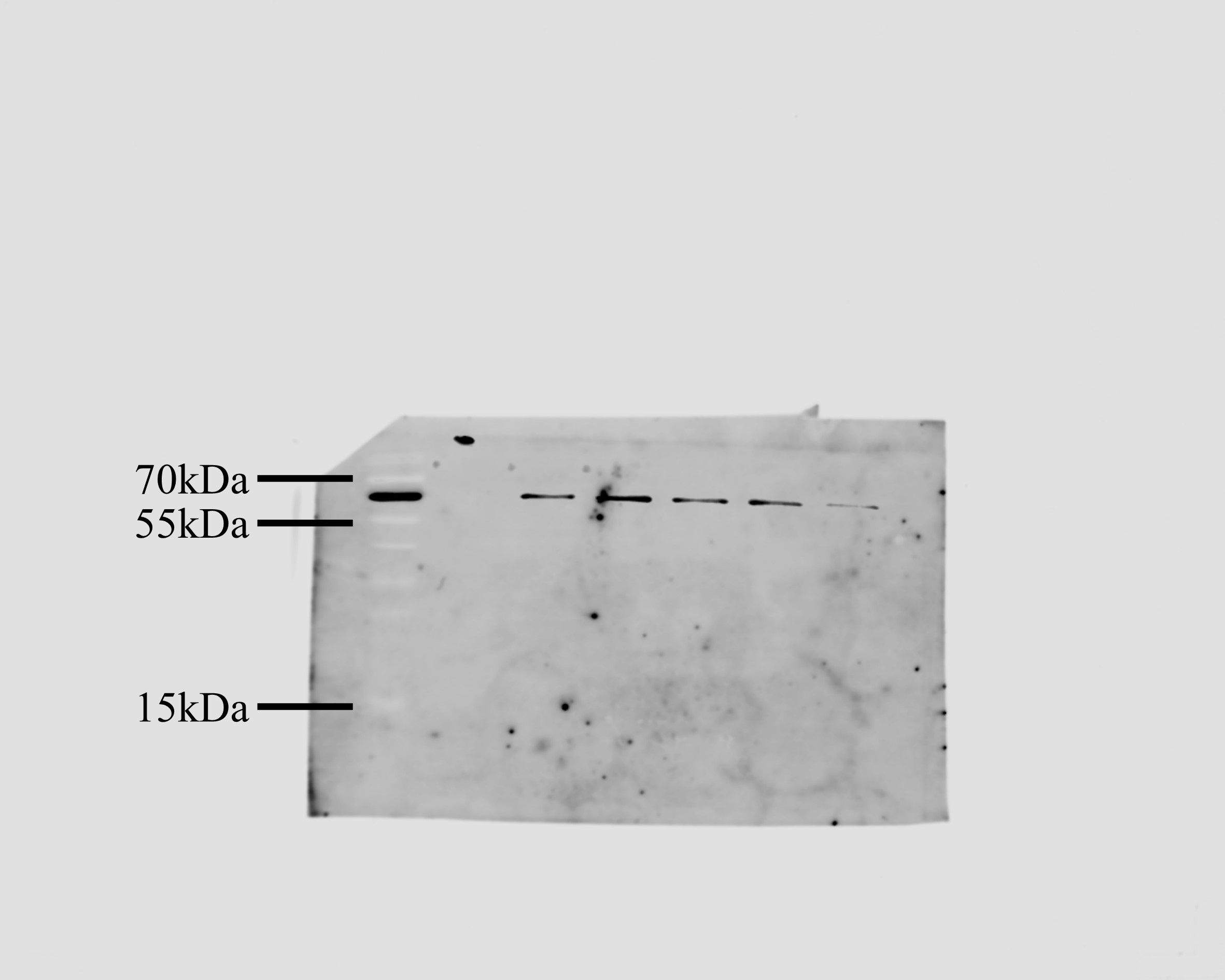

Supplement: Supplementary file 4 — Supplementary Information 4. [file 41598_2024_61892_MOESM4_ESM.jpg]

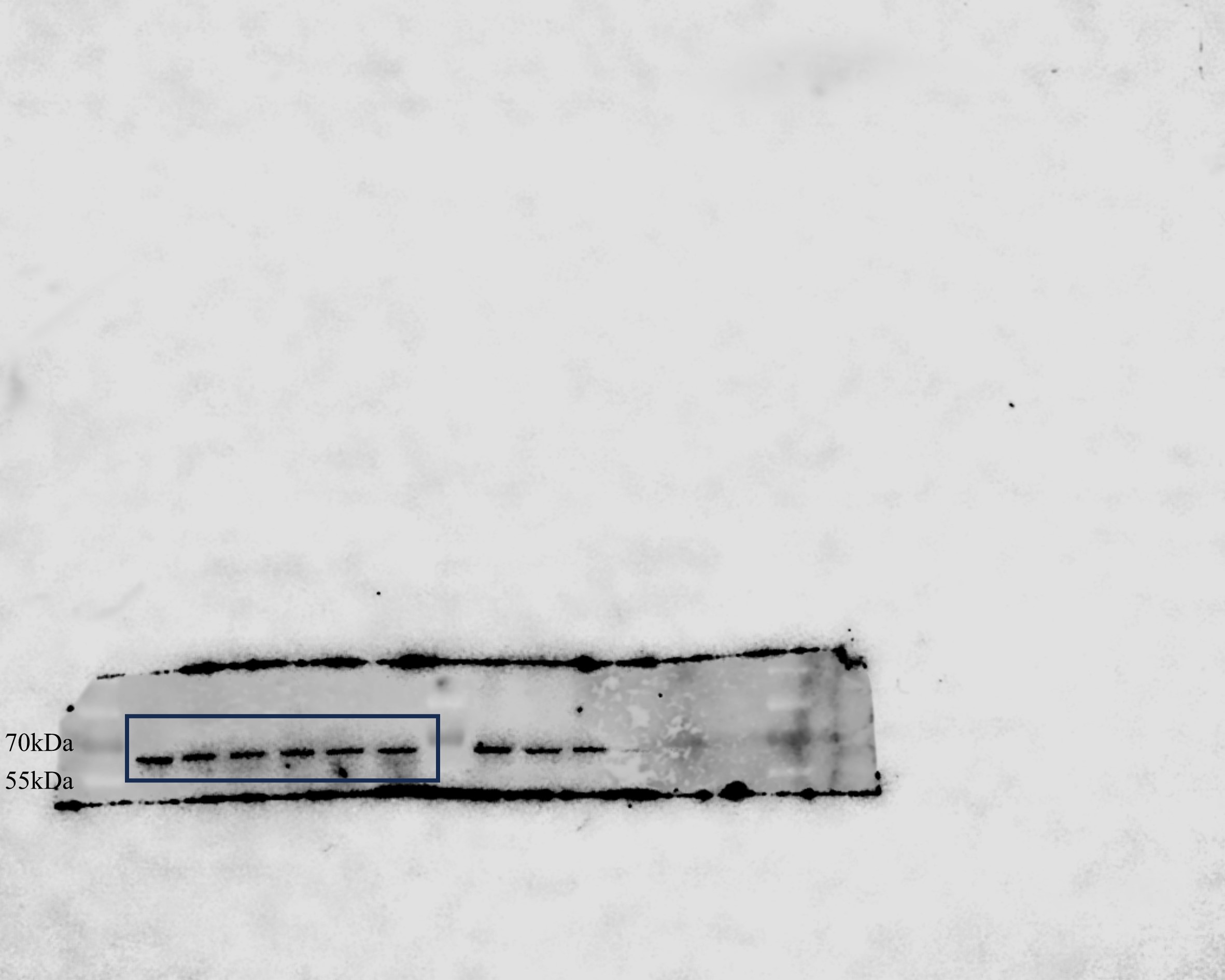

Supplement: Supplementary file 5 — Supplementary Information 5. [file 41598_2024_61892_MOESM5_ESM.jpg]

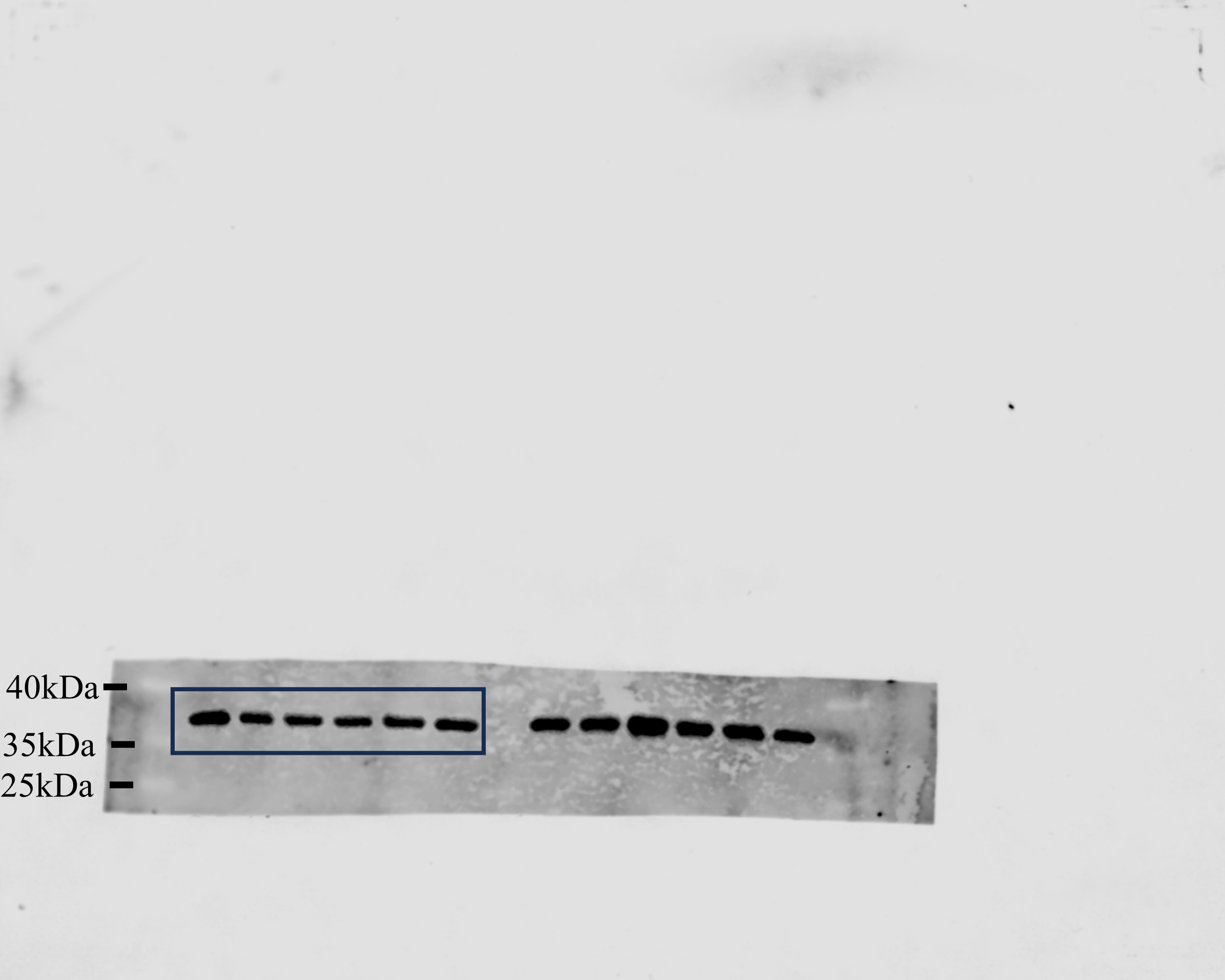

Supplement: Supplementary file 6 — Supplementary Information 6. [file 41598_2024_61892_MOESM6_ESM.jpg]

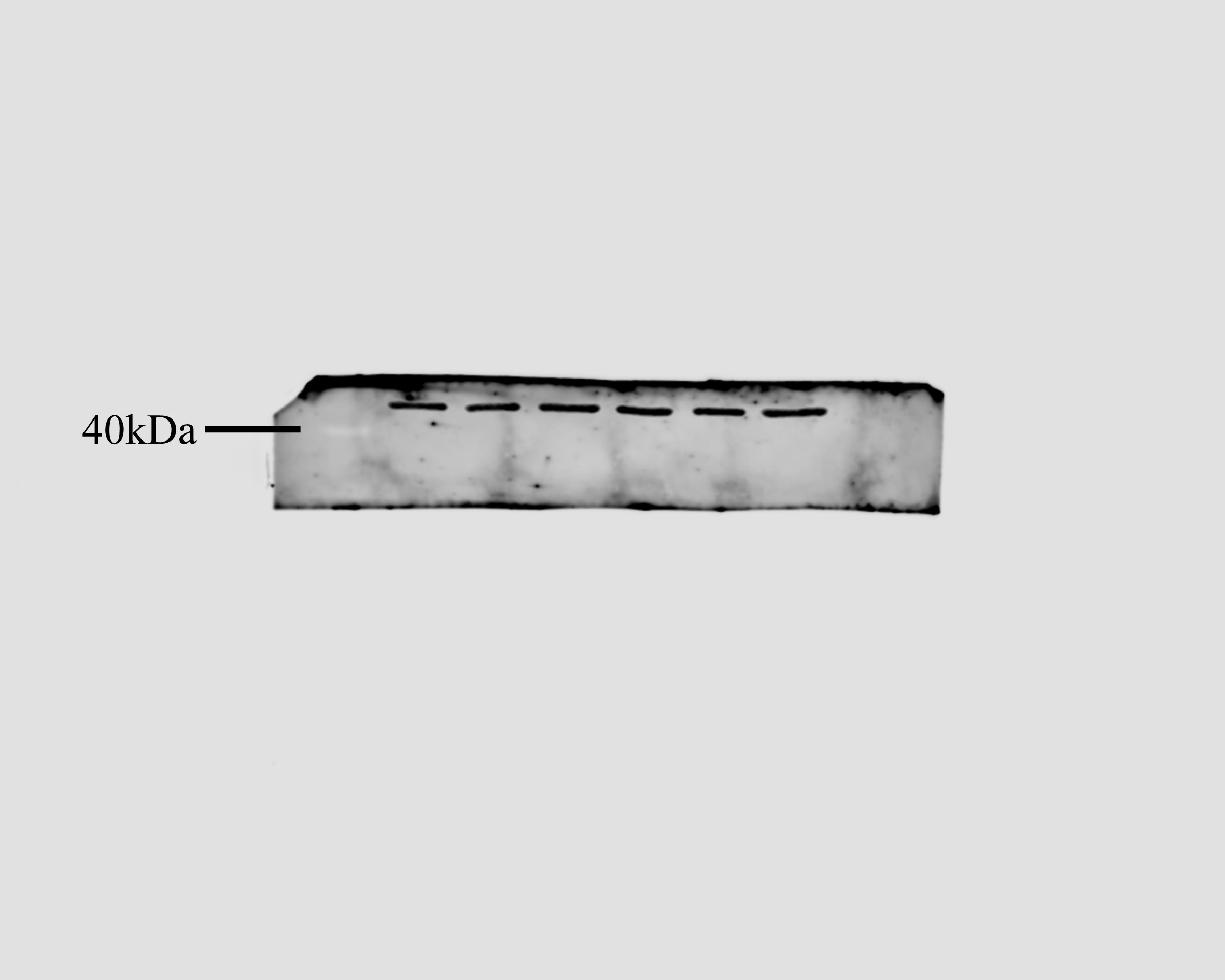

Supplement: Supplementary file 7 — Supplementary Information 7. [file 41598_2024_61892_MOESM7_ESM.jpg]

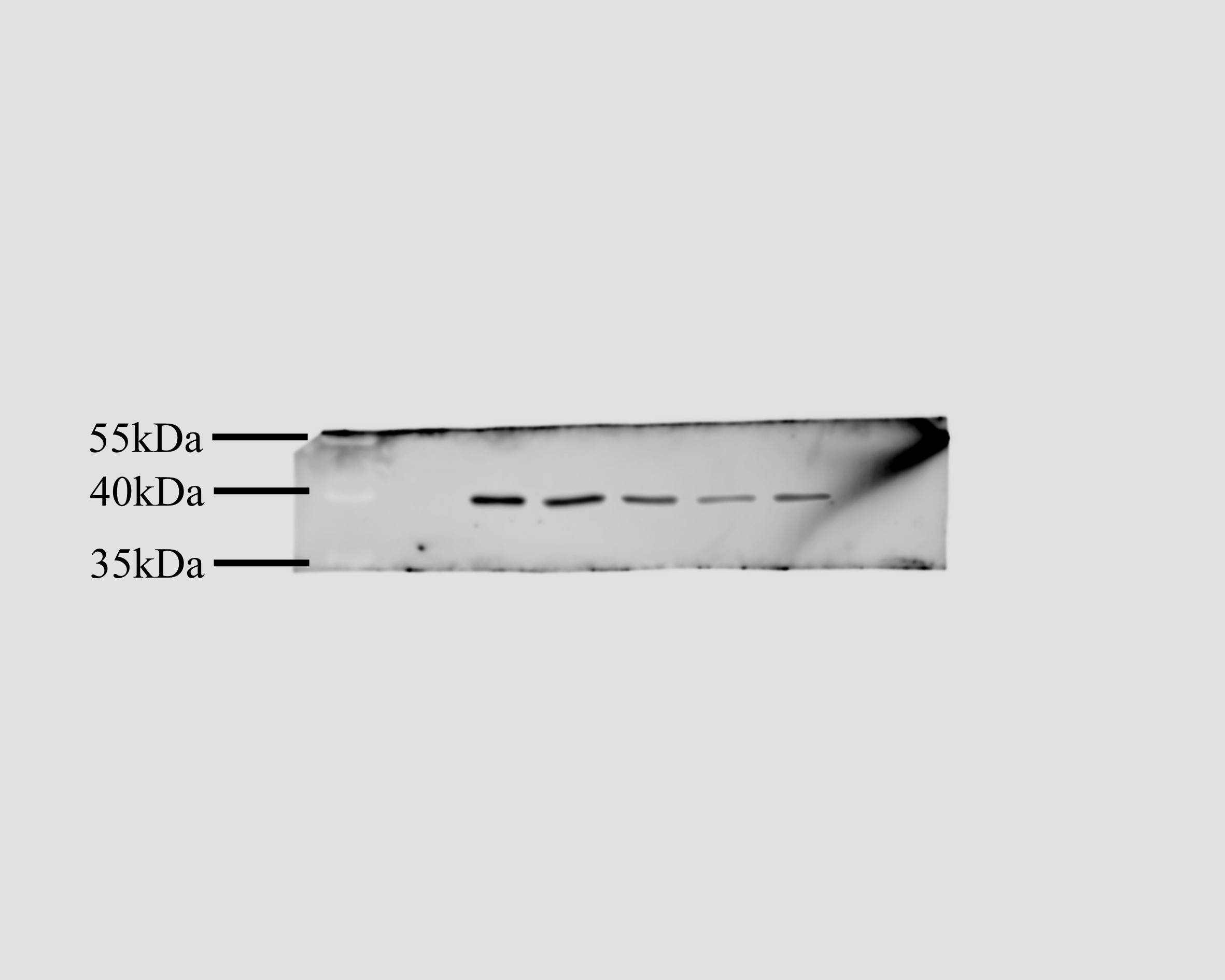

Supplement: Supplementary file 8 — Supplementary Information 8. [file 41598_2024_61892_MOESM8_ESM.jpg]

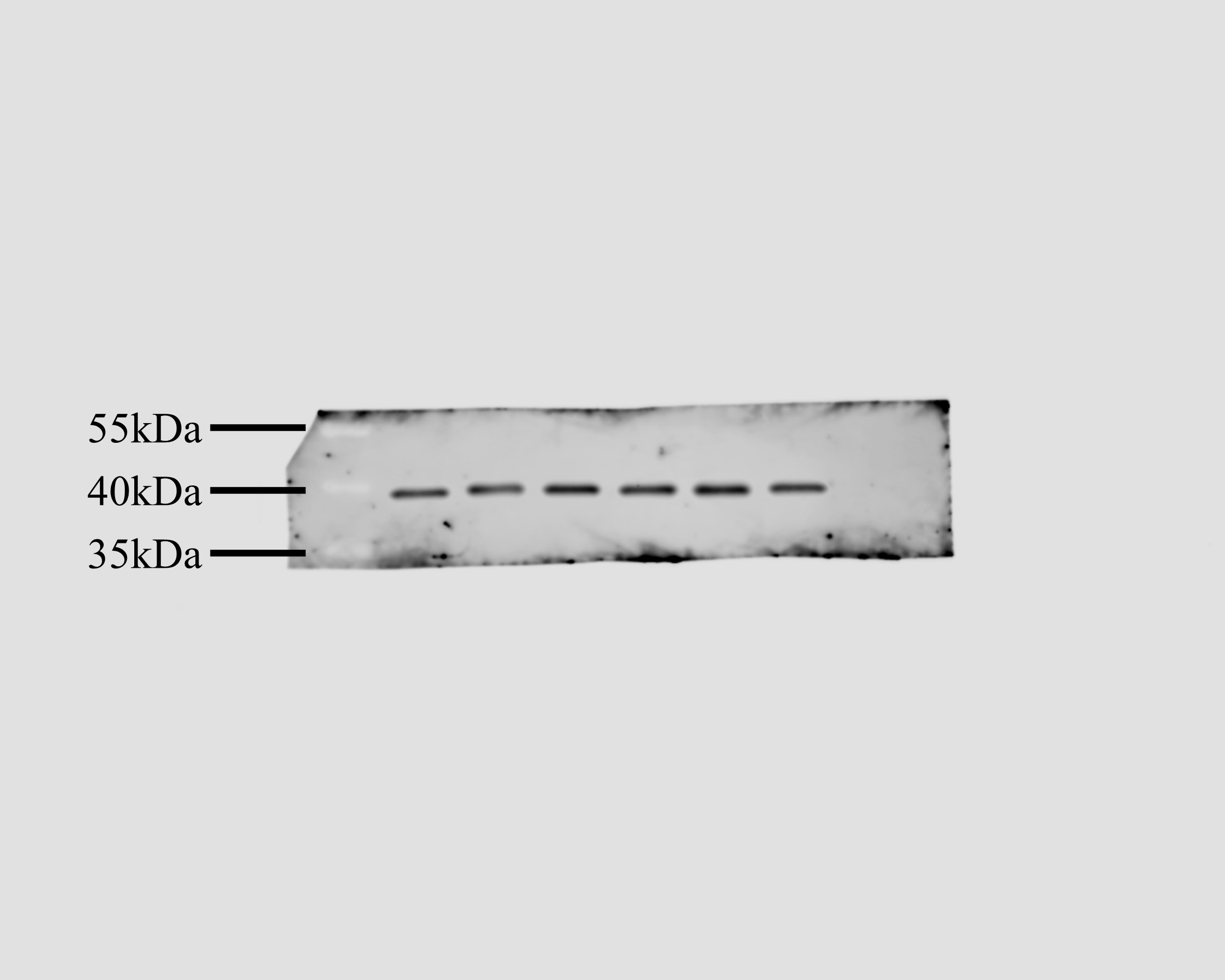

Supplement: Supplementary file 9 — Supplementary Information 9. [file 41598_2024_61892_MOESM9_ESM.jpg]

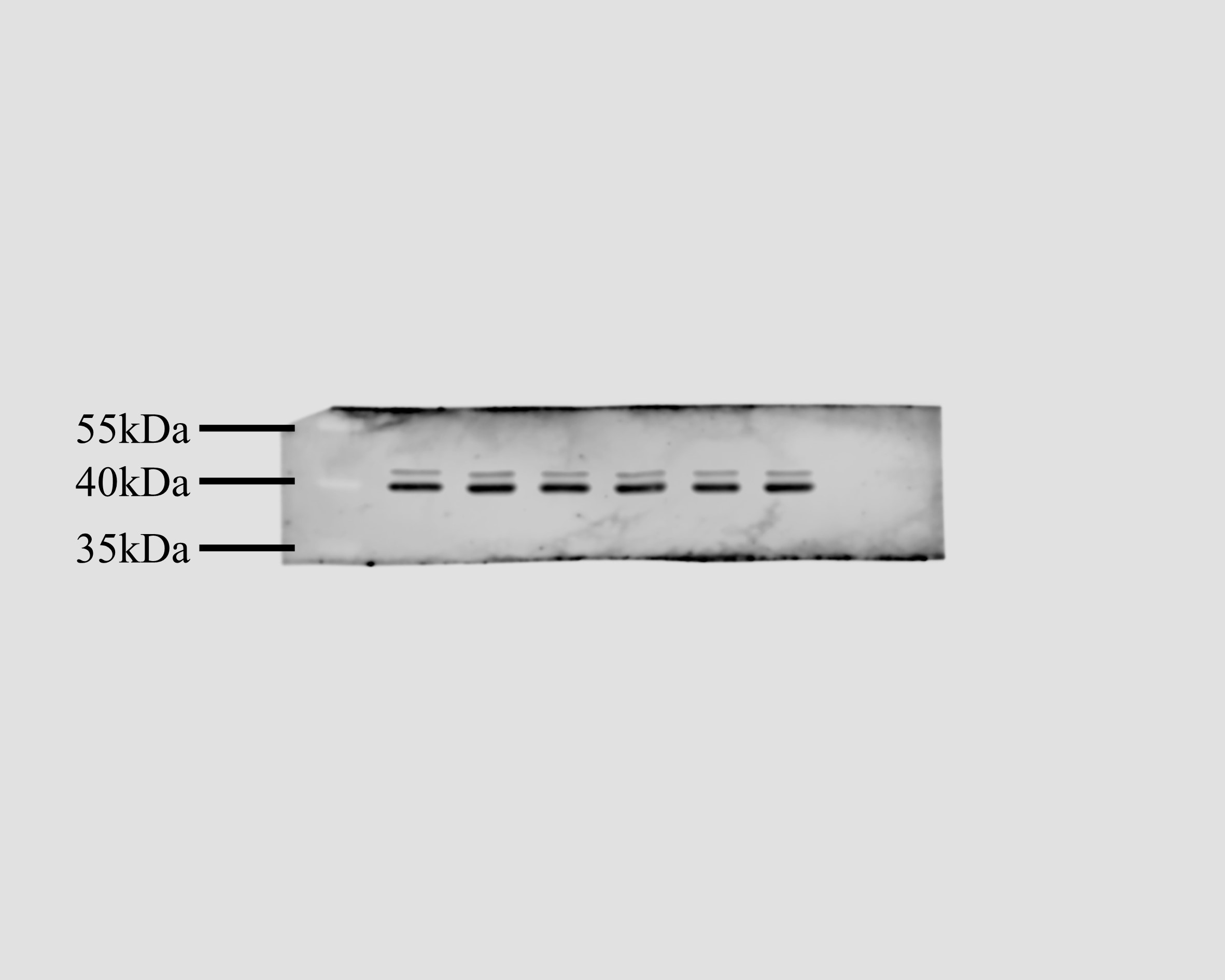

Supplement: Supplementary file 10 — Supplementary Information 10. [file 41598_2024_61892_MOESM10_ESM.jpg]

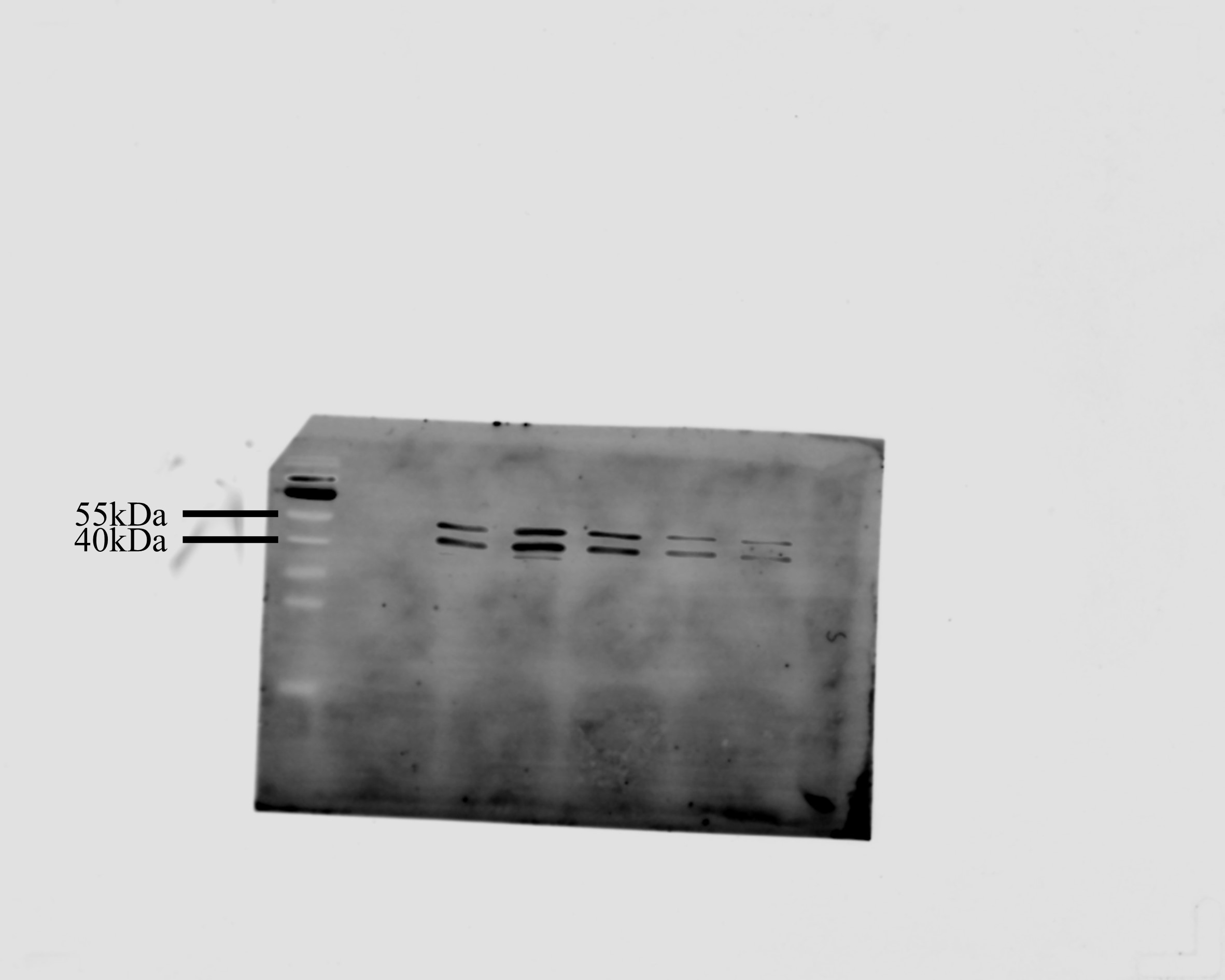

Supplement: Supplementary file 12 — Supplementary Information 12. [file 41598_2024_61892_MOESM12_ESM.jpg]

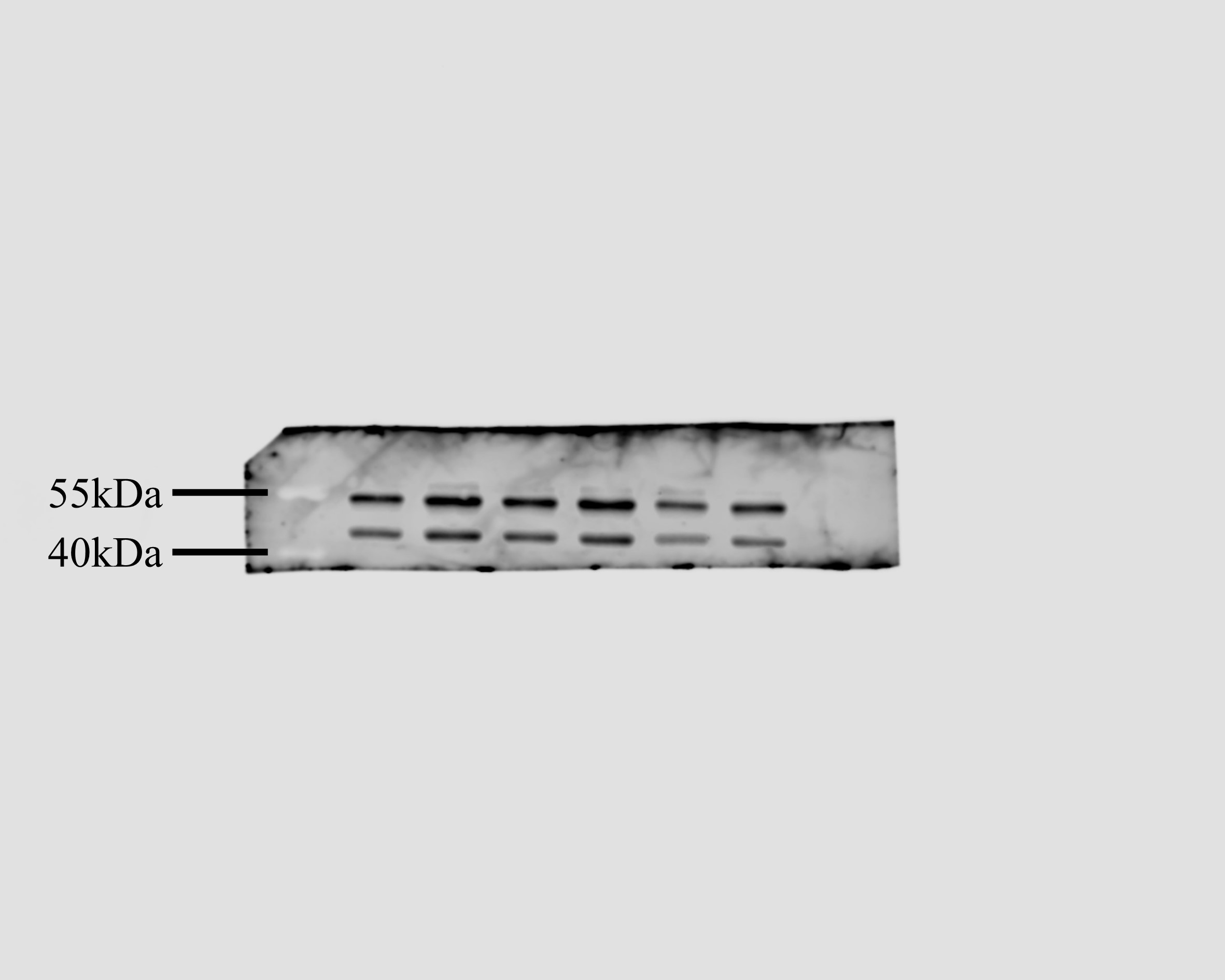

Supplement: Supplementary file 13 — Supplementary Information 13. [file 41598_2024_61892_MOESM13_ESM.jpg]

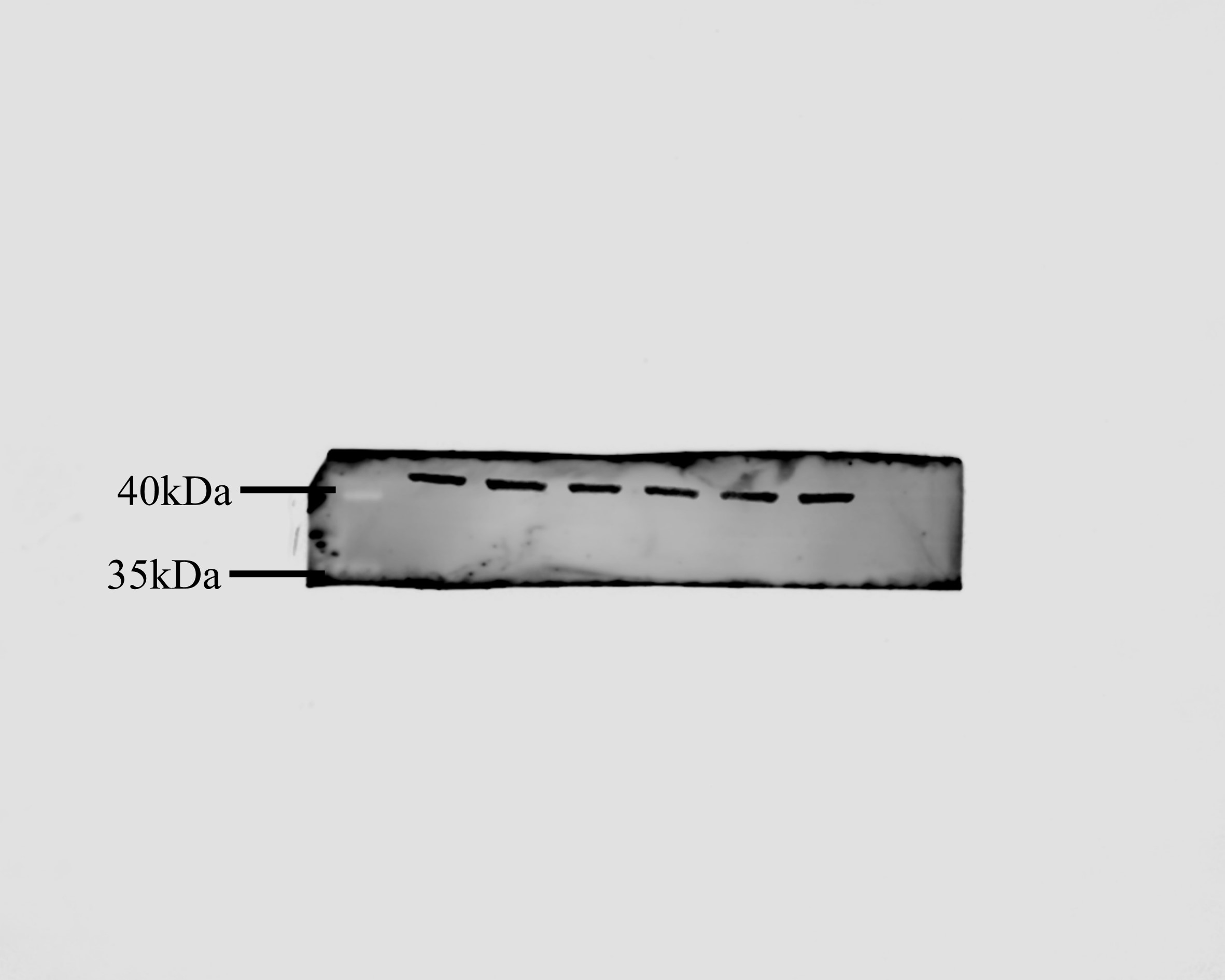

Supplement: Supplementary file 14 — Supplementary Information 14. [file 41598_2024_61892_MOESM14_ESM.jpg]

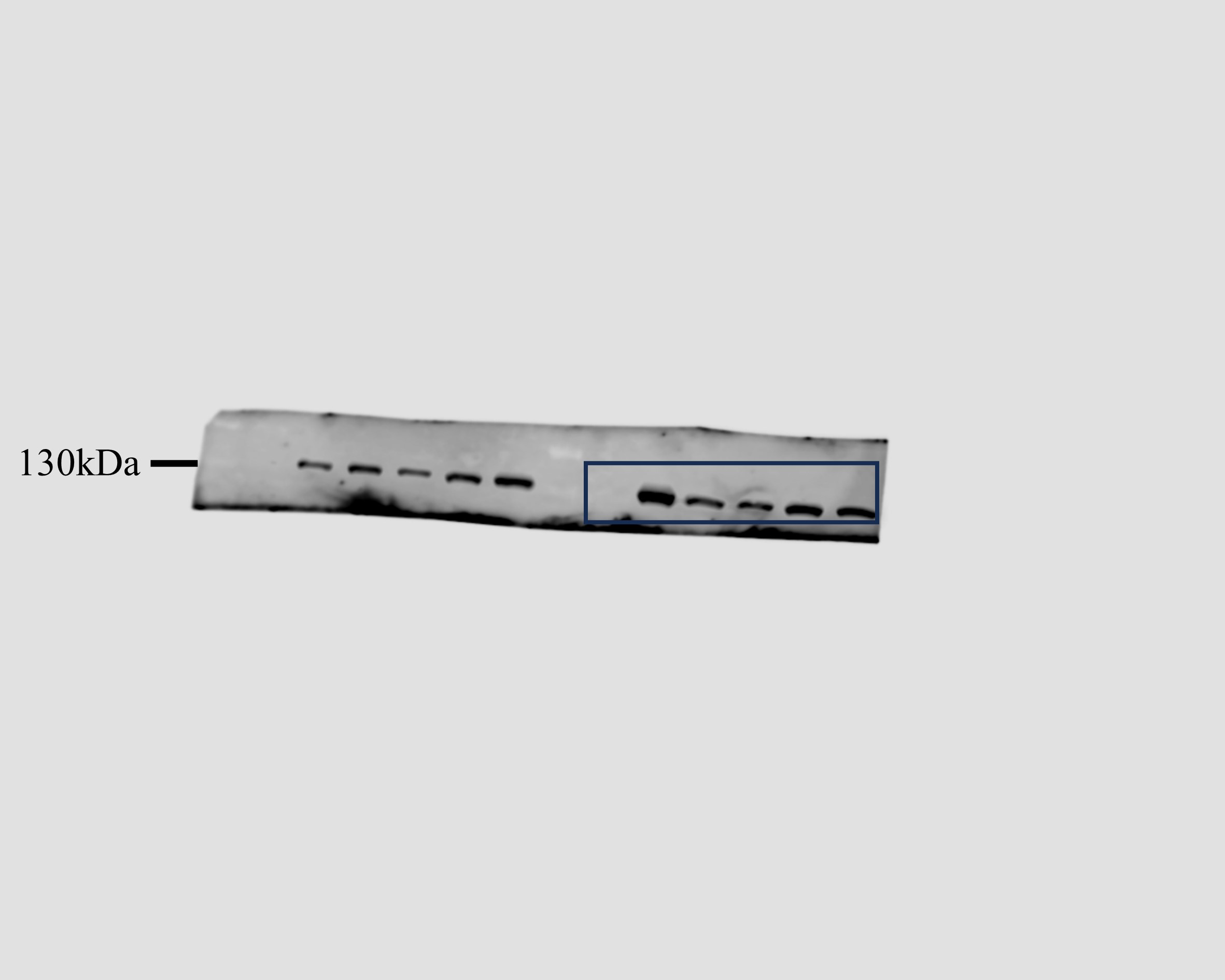

Supplement: Supplementary file 15 — Supplementary Information 15. [file 41598_2024_61892_MOESM15_ESM.jpg]

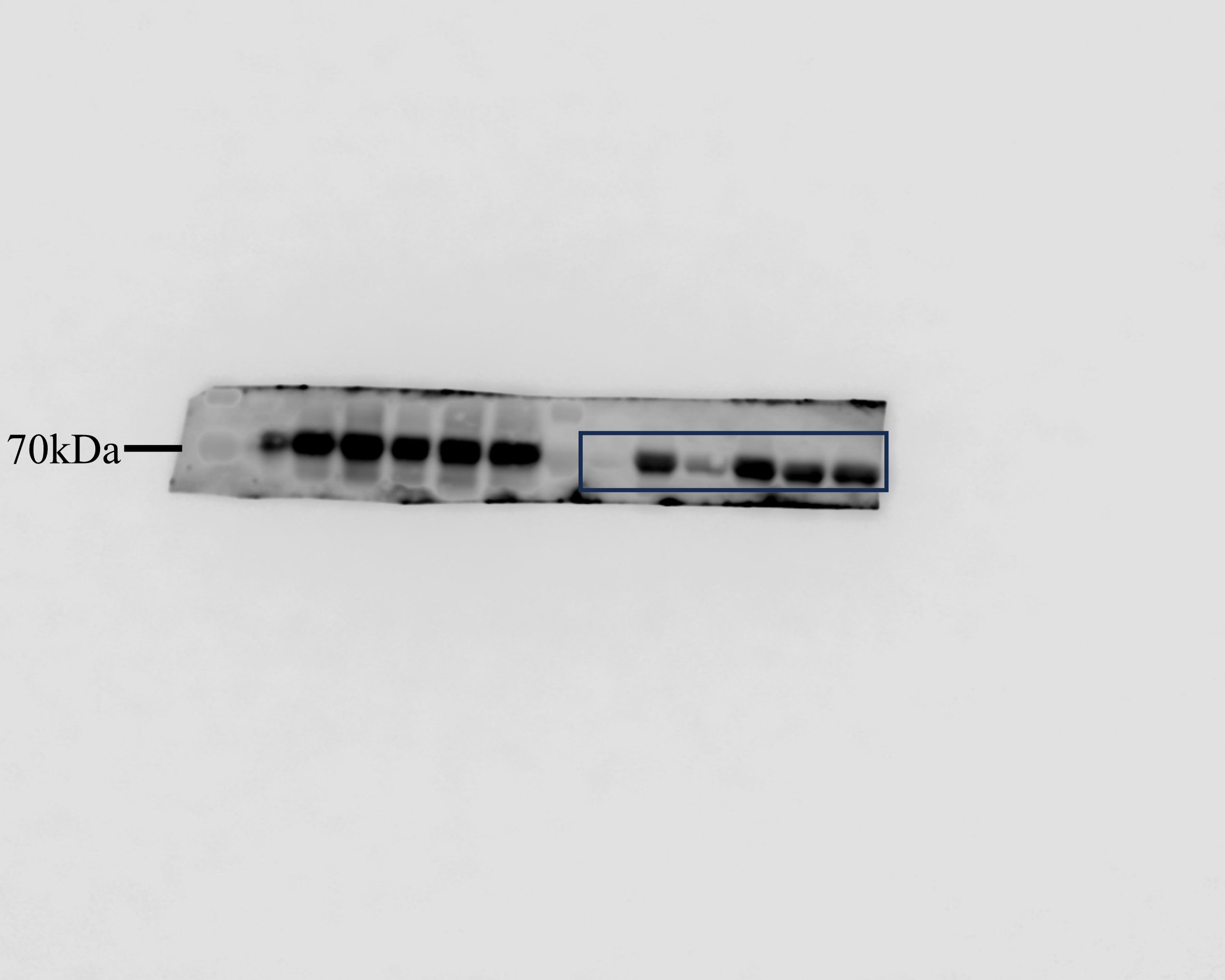

Supplement: Supplementary file 16 — Supplementary Information 16. [file 41598_2024_61892_MOESM16_ESM.jpg]

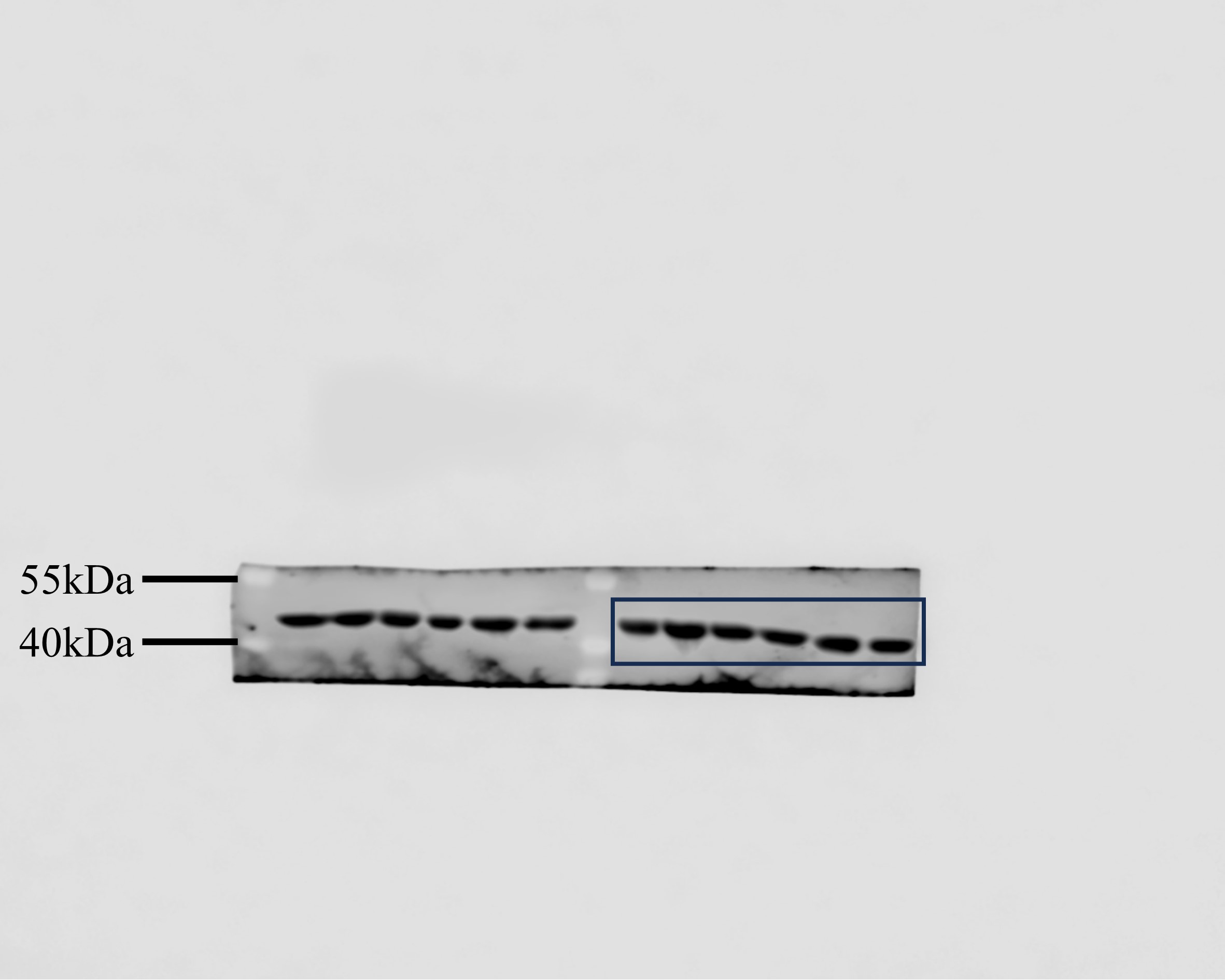

Supplement: Supplementary file 17 — Supplementary Information 17. [file 41598_2024_61892_MOESM17_ESM.jpg]
