# Supplementary material for: Anti-inflammatory properties and characterization of water extracts obtained from Callicarpa kwangtungensis Chun using in vitro and in vivo rat models
Source: Sci Rep. 2024 May 14;14:11047. doi: 10.1038/s41598-024-61892-9 (PMC11094131; doi:10.1038/s41598-024-61892-9)
Supplement: Supplementary file 18 — Supplementary Information 18. [file 41598_2024_61892_MOESM18_ESM.docx]

**Table 1**

Identification of 68 compounds from *Callicarpa kwangtungensis* Chun water extract

| No. | T_R_ (min) | Identification | Category | Formula | Ion mode | Theo. Mass  (m/z) | Obser. Mass (m/z) | Error  (ppm) | MS/MS fragments (m/z) |
| --- | --- | --- | --- | --- | --- | --- | --- | --- | --- |
| 1 | 3.12 | Coniferaldehyde | Phenols | C_10_H_10_O_3_ | [M+H]^+^ | 179.07027 | 179.07021 | -0.339 | 179.07021[M+H]^+^, 151.07547[M+H-CO]^+^, 147.04399[M+H-CH_2_-H_2_O]^+^,119.04932[M+H-CH_2_-H_2_O-CO]^+^ |
| 2 | 4.01 | 2-hydroxyl-5-methoxybenzoic acid | Organic acids | C_8_H_8_O_4_ | [M-H]^-^ | 167.03498 | 167.03362 | -1.588 | 167.03362[M-H]^-^, 149.02303[M-H-H_2_O]^-^, 137.02299[M-H-OCH_2_]^-^, 123.04361[M-H-CO_2_]^-^, 93.03303[M-H-OCH_2_-CO_2_]^-^ |
| 3 | 4.37 | 3,4,5-trimethoxyphenyl-β-D-glucopyranoside | Phenols | C_15_H_22_O_9_ | [M+H]^+^ | 347.13366 | 347.13113 | -7.285 | 347.13150[M+H]^+^, 285.13098[M+H-2OCH_3_]^+^ |
| 4 | 4.89 | Vanillic acid | Organic acids | C_8_H_8_O_4_ | [M-H]^-^ | 167.03498 | 167.03365 | -1.408 | 167.03365[M-H]^-^, 152.01003[M-H-CH_3_]^-^, 123.04365[M-H-CO_2_]^-^, 108.02012[M-H-CH_3_-CO_2_]^-^, 93.03328[M-H-OCH_2_-CO_2_]^-^ |
| 5 | 5.55 | Forsythoside E | Phenylethanoid glycosides | C_20_H_30_O_12_ | [M-H]^-^ | 461.16645 | 461.16504 | -0.678 | 461.16504[M-H]^-^, 315.10870[M-H-Rha]^-^, 153.05437[phenethanol]^-^, 135.04364[phenethanol-H_2_O]^-^ |
| 5 | 5.56 |  |  |  | [M+H]^+^ | 463.181 | 463.18076 | -0.524 | 463.18076[M+H]^+^, 301.07050[M+H-ORha]^+^, 286.04700[M+H-Rha-CH_3_]^+^, 258.05212[M+H-Rha-CH_3_-CO]^+^ |
| 6 | 5.79 | Peiioside A | Organic acids | C_26_H_36_O_17_ | [M-H]^-^ | 619.18797 | 619.18634 | -2.640 | 619.18634[M-H]^-^, 179.03365[caffeic acid-H]^-^, 161.02303[caffeic acid-H-H_2_O]^-^, 135.04367[caffeic acid-H-CO_2_]^-^ |
| 7 | 6.56 | 3,4-dihydroxybenzoic acid | Organic acids | C_7_H_6_O_4_ | [M-H]^-^ | 153.01933 | 153.01788 | -2.321 | 153.01788[M-H]^-^, 109.02794[M-H-CO_2_]^-^ |
| 8 | 6.56 | Orthoanisic acid | Organic acids | C_8_H_8_O_3_ | [M-H]^-^ | 151.04007 | 151.03867 | -9.252 | 151.03867[M-H]^-^, 136.01508[M-H-CH_3_]^-^ |
| 9 | 6.73 | Caffeic acid | Organic acids | C_9_H_8_O_4_ | [M-H]^-^ | 179.03498 | 179.03362 | -1.481 | 179.03362[M-H]^-^, 135.04366[M-H-CO_2_]^-^ |
| 10 | 6.78 | Pinnatifidanoid A | Terpenoids | C_13_H_24_O_3_ | [M+H]^+^ | 229.17982 | 229.17966 | -0.703 | 229.17987[M+H]^+^, 211.16962[M+H-H_2_O]^+^, 193.12270[M+H-2H_2_O]^+^ |
| 11 | 6.79 | 4-hydroxy cinnamic aicd | Organic acids | C_9_H_8_O_3_ | [M-H]^-^ | 163.04007 | 163.03865 | -1.966 | 163.03865[M-H]^-^, 145.02817[M-H-H_2_O]^-^, 135.04369[M-H-CO]^-^, 119.04860[M-H-CO_2_]^-^ |
| 12 | 6.79 | Methoxyquinol4-O-[(5-O-trans-p-caffeoyl)-β-D-apiofuranosyl-(1→2)-β-D-glucopyranoside] | Organic acids | C_27_H_32_O_15_ | [M-H]^-^ | 595.16684 | 595.16559 | -0.157 | 595.16559[M-H]^-^, 387.14487[M-H-caffeoyl-HCOOH]^-^, 357.09583[M-caffeoyl-HCOOH-OCH_3_]^-^, 301.07147[M-H-caffeoyl-Api]^-^ |
| 13 | 6.84 | Cistanoside F | Organic acids | C_21_H_28_O_13_ | [M-H]^-^ | 487.14571 | 487.14453 | -2.431 | 487.14453[M-H]^-^, 179.03360[caffeic acid-H]^-^, 161.02301[caffeic acid-H-H_2_O]^-^, 135.04366[caffeic acid-H-CO_2_]^-^ |
| 14 | 7.06 | Daidzin | Flavonoids | C_21_H_20_O_9_ | [M-H]^-^ | 415.10346 | 415.10226 | -0.356 | 415.10226[M-H]^-^, 295.06036[M-H-C_8_H_8_O]^-^, 267.06555[M-H-C_8_H_8_O-CO]^-^, 253.04932[M-H-Glc]^-^ |
| 14 | 7.07 |  |  |  | [M+H]^+^ | 417.11801 | 417.11786 | -0.356 | 417.11786[M+H]^+^, 399.10654[M+H-H_2_O]^+^, 381.09741[M+H-2H_2_O]^+^, 297.07556[M+H-C_8_H_4_O]^+^ |
| 15 | 7.16 | Tuberonic acid glucoside | Organic acids | C_18_H_28_O_9_ | [M-H]^-^ | 387.16606 | 387.16492 | -2.933 | 387.16525[M-H]^-^, 207.10179[M-H-Glc-H_2_O]^-^, 163.11159[M-H-Glc-H_2_O-CO_2_]^-^ |
| 15 | 7.17 |  |  |  | [M+H]^+^ | 389.18061 | 389.18011 | -1.282 | 389.18011[M+H]^+^, 227.16446[M+H-Glc]^+^, 209.15361[M+H-Glc-H_2_O]^+^, 191.14284[M+H-Glc-2H_2_O]^+^, 167.07027[M+3H-Glc-H_2_O-CO_2_]^+^ |
| 16 | 7.49 | Melilotoside | Organic acids | C_15_H_18_O_8_ | [M-H]^-^ | 325.09289 | 325.09207 | 0.849 | 325.09207[M-H]^-^, 179.05475[Glc]^-^, 163.03868[M-H-Glc]^-^, 145.02786[M-H-Glc-H_2_O]^-^ |
| 17 | 7.61 | Vanillin | Phenols | C_8_H_8_O_3_ | [M-H]^-^ | 151.04007 | 151.03867 | -1.990 | 151.03984[M-H]^-^, 136.01637[M-H-CH_3_]^-^, 133.02892[M-H-H_2_O]^-^, 123.04494[M-H-CO]^-^, 108.02151[M-H-CH_3_-CO]^-^ |
| 17 | 7.66 |  |  |  | [M+H]^+^ | 153.05462 | 153.05457 | -0.331 | 153.05457[M+H]^+^, 135.11690[M+H-H_2_O]^+^, 125.09639[M+H-CO]^+^ |
| 18 | 7.65 | Syringic acid | Organic acids | C_9_H_10_O_5_ | [M-H]^-^ | 197.04555 | 197.04427 | -0.913 | 197.04427[M-H]^-^, 182.02080[M-H-CH_3_]^-^, 166.99710[M-H-2CH_3_]^-^, 153.05426[M-H-CO_2_]^-^, 123.00737[M-H-2CH_3_-CO_2_]^-^ |
| 19 | 7.78 | β-OH-poliumoside | Phenylethanoid glycosides | C_35_H_46_O_20_ | [M-H]^-^ | 785.25097 | 785.24927 | -0.764 | 785.25000[M-H]^-^, 179.03380[caffeic acid]-, 161.02316[caffeic acid-H_2_O]^-^ |
| 20 | 7.80 | Echinacoside | Phenylethanoid glycosides | C_35_H_46_O_20_ | [M-H]^-^ | 785.25097 | 785.24927 | -0.764 | 785.25122[M-H]^-^, 623.20190[M-H-caffeoyl]^-^, 461.16641[M-H-caffeoyl-Glc]^-^, 315.10880[M-H-caffeoyl-Glc-Rha]^-^, 179.03473[caffeic acid]^-^, 161.02428[caffeic acid-H_2_O]^-^, 135.04503[caffeic acid-CO_2_]^-^, 153.05525[phenethanol]^-^ |
| 21 | 8.41 | Samioside | Phenylethanoid glycosides | C_34_H_44_O_19_ | [M-H]^-^ | 755.2404 | 755.23816 | -1.516 | 755.23816[M-H]^-^, 623.19830[M-H-Api]^-^, 593.20947[M-H-caffeoyl]^-^, 447.14832[M-H-caffeoyl-Rha]^-^, 179.03371[caffeic acid]-, 161.02295[caffeic acid-H_2_O]^-^ |
| 22 | 8.69 | 5-methylisophthalic acid monomethyl ester | Organic acids | C_10_H_10_O_4_ | [M-H]^-^ | 193.05063 | 193.04936 | -0.908 | 193.04945[M-H]^-^, 178.02612[M-H-CH_3_]^-^, 149.05951[M-H-CO_2_]^-^ |
| 23 | 8.80 | Poliumoside | Phenylethanoid glycosides | C_35_H_46_O_19_ | [M-H]^-^ | 769.25605 | 769.25409 | -1.125 | 769.25482[M-H]^-^, 607.22351[M-H-caffeoyl]^-^, 461.16376[M-H-caffeoyl-Rha]^-^, 179.03383[caffeic acid]^-^, 161.02319[caffeic acid-H_2_O]^-^, 135.04385[caffeic acid-CO_2_]^-^ |
| 24 | 8.81 | Luteolin 3'-O-glucuronide | Flavonoids | C_21_H_18_O_12_ | [M+H]^+^ | 463.0871 | 463.08676 | -0.739 | 463.08676[M+H]^+^, 287.05493[M+H-GlcA]^+^, 269.04337[M+H-GlcA-H_2_O]^+^, 153.01823[M+H-GlcA-C_8_H_6_O_2_]^+^ |
| 24 | 8.84 |  |  |  | [M-H]^-^ | 461.07255 | 461.07138 | -0.157 | 461.07162[M-H]^-^, 285.03983[M-H-GlcA]^-^, 151.00243[M-H-C_8_H_6_O_2_]^-^ |
| 25 | 8.86 | Acteoside | Phenylethanoid glycosides | C_29_H_36_O_15_ | [M-H]^-^ | 623.19814 | 623.19647 | -0.925 | 623.19696[M-H]^-^, 461.16580[M-H-caffeoyl]^-^, 315.10794[M-H-caffeoyl-Rha]^-^, 179.03384[caffeic acid]^-^, 161.02310[caffeic acid-H_2_O]^-^, 135.04375[caffeic acid-CO_2_]^-^ |
| 26 | 8.91 | Forsythoside B | Phenylethanoid glycosides | C_34_H_44_O_19_ | [M-H]^-^ | 755.2404 | 755.23822 | -1.437 | 755.23834[M-H]^-^, 593.20703[M-H-caffeoyl]^-^, 461.16846[M-H-caffeoyl-Api]^-^, 315.10757[M-H-caffeoyl-Api-Rha]^-^, 179.03371[caffeic acid-H]^-^, 161.02303[caffeic acid-H-H_2_O]^-^ |
| 27 | 9.10 | Alyssonoside | Phenylethanoid glycosides | C_35_H_46_O_19_ | [M-H]^-^ | 769.25605 | 769.25403 | -1.203 | 769.25482[M-H]^-^, 593.20795[M-H-feruloyl]^-^, 461.16589[M-H-feruloyl-Api]^-^, 175.03886[feruloyl-H_2_O]^-^, 161.02309[feruloyl-CH_3_OH]^-^ |
| 28 | 9.25 | Isoacteoside | Phenylethanoid glycosides | C_29_H_36_O_15_ | [M-H]^-^ | 623.19814 | 623.19641 | -1.022 | 623.19708[M-H]^-^, 461.16574[M-H-caffeoyl]^-^, 315.10867[M-H-caffeoyl-Rha]^-^, 179.03374[caffeic acid]^-^, 161.02313[caffeic acid-H_2_O]^-^, 135.04373[caffeic acid-CO_2_]^-^ |
| 29 | 9.26 | Loliolide | Terpenoids | C_11_H_16_O_3_ | [M+H]^+^ | 197.11722 | 197.1172 | -0.106 | 197.11745[M+H]^+^, 179.10667[M+H-H_2_O]^+^, 161.09633[M+H-2H_2_O]^+^, 151.03914[M+H-H_2_O-CO]^+^ |
| 30 | 9.47 | 2'-acetyl forsythoside B | Phenylethanoid glycosides | C_36_H_46_O_20_ | [M-H]^-^ | 797.25097 | 797.24915 | -0.903 | 797.24915[M-H]^-^, 593.20795[M-H-CO-CH_2_-caffeoyl]^-^, 179.03372[caffeic acid]^-^, 161.02298[caffeic acid-H_2_O]^-^ |
| 31 | 9.49 | Longissimoside B | Phenylethanoid glycosides | C_36_H_48_O_19_ | [M-H]^-^ | 783.2717 | 783.2699 | -0.901 | 783.27008[M-H]^-^, 607.22333[M-H-feruloyl]^-^, 461.16553[M-H-feruloyl-Rha]^-^, 193.04951[feruloyl]^-^, 175.03883[feruloyl-H_2_O]^-^ |
| 32 | 9.53 | Chrisin-6,8-C-glycosides | Flavonoids | C_27_H_30_O_14_ | [M+H]^+^ | 579.17083 | 579.17041 | -0.437 | 579.17041[M+H]^+^, 433.11313[M+H-C_9_H_6_O_2_]^+^, 271.05997[M+H-C_9_H_6_O_2_-Glc]^+^, 243.06554[M+H-C_9_H_6_O_2_-Glc-CO]^+^ |
| 33 | 9.54 | Dehydroxy forsythoside B | Phenylethanoid glycosides | C_34_H_44_O_18_ | [M-H]^-^ | 739.24549 | 739.24396 | -0.583 | 739.24396[M-H]^-^, 577.21460[M-H-caffeoyl]^-^, 445.17410[M-H-caffeoyl-Api]^-^, 179.03386[caffeic acid]^-^, 161.02299[caffeic acid-H_2_O]^-^ |
| 34 | 9.61 | Genistin | Flavonoids | C_21_H_20_O_10_ | [M+H]^+^ | 433.11292 | 433.1127 | -0.515 | 433.11328[M+H]^+^, 271.05963[M+3H-Glc]^+^, 153.01826[M+3H-Glc-C_8_H_6_O]^+^, 363.08603[M+H-3H_2_O]^+^, 297.07559[M+H-C_8_H_4_O]^+^ |
| 35 | 9.66 | Apigenin-7-glucuronide | Flavonoids | C_21_H_18_O_11_ | [M+H]^+^ | 447.09219 | 447.09192 | -0.599 | 447.09219[M+H]^+^, 271.05978[M+H-GlcA]^+^, 153.01811[M+H-GlcA-C_8_H_6_O]^+^ |
| 35 |  |  |  |  | [M-H]^-^ | 445.07763 | 445.07648 | -0.130 | 445.07648[M-H]^-^, 269.04477[M-H-GlcA]^-^, 225.05428[M-H-GlcA-CO_2_]^-^ |
| 36 | 9.77 | Blumenol C | Terpenoids | C_13_H_22_O_2_ | [M+H]^+^ | 211.16926 | 211.16899 | -1.262 | 211.16899[M+H]^+^, 193.15814[M+H-H_2_O]^+^, 181.08562[M+H-2CH_3_]^+^ |
| 37 | 9.84 | Syringalide A 3'-α-l-rhamnopyranoside | Phenylethanoid glycosides | C_29_H_36_O_14_ | [M-H]^-^ | 607.20323 | 607.20178 | -0.580 | 607.20258[M-H]^-^, 461.16644[M-H-Rha]^-^, 179.03380[caffeic acid]^-^, 161.02313[caffeic acid-H_2_O]^-^ |
| 38 | 9.97 | Cynaroside | Flavonoids | C_21_H_20_O_11_ | [M-H]^-^ | 447.09328 | 447.09222 | -2.381 | 447.09326[M-H]^-^, 285.04025[M-H-Glc]^-^, 256.03781[M-H-Glc-CHO]^-^, 151.00343[M-H-C_8_H_6_O_2_]^-^, 107.01363[M-H-C_8_H_6_O_2_-CO_2_]^-^ |
| 39 | 10.08 | Chrysoeriol-7-O-β-D-glucopyranoside | Flavonoids | C_22_H_22_O_11_ | [M-H]^-^ | 461.10893 | 461.23822 | -2.169 | 461.10834[M-H]^-^, 446.08459[M-H-CH_3_]^-^, 298.04758[M-H-Glc]^-^, 283.02429[M-H-Glc-CH_3_]^-^, 255.02917[M-H-Glc-CH_3_-CO]^-^ |
| 40 | 10.21 | Ferulic acid | Organic acids | C_10_H_10_O_4_ | [M-H]^-^ | 193.05063 | 193.04939 | -0.753 | 193.04959[M-H]^-^, 178.02618[M-H-CH_3_]^-^, 149.05936[M-H-CO_2_]^-^ |
| 41 | 10.32 | O-(methoxycarbonyl) phenylacetic acid | Organic acids | C_10_H_10_O_4_ | [M-H]^-^ | 193.05063 | 193.04959 | -0.753 | 193.04959[M-H]^-^, 178.02623[M-H-CH_3_]^-^ |
| 42 | 10.48 | Philonotisflavone | Flavonoids | C_30_H_26_O_14_ | [M-H]^-^ | 609.12498 | 609.20703 | -1.114 | 609.12415[M-H]^-^, 447.10767[M-H-caffeoyl]^-^, 285.03992[M-H-caffeoyl-Glc]^-^, 151.07516[M-H-caffeoyl-Glc-C_8_H_6_O_2_]^-^ |
| 43 | 10.48 | 2'-acetylacteoside | Phenylethanoid glycosides | C_31_H_38_O_16_ | [M-H]^-^ | 665.20871 | 665.20709 | -0.784 | 665.20709[M-H]^-^, 315.10803[M-H-caffeoyl-COCH_2_-Rha]^-^, 179.03378[caffeic acid]^-^, 161.02293[caffeic acid-H_2_O]^-^ |
| 44 | 10.95 | Daidzein | Flavonoids | C_15_H_10_O_4_ | [M+H]^+^ | 255.06519 | 255.06493 | -1.001 | 255.06499[M+H]^+^, 227.07013[M+H-CO]^+^, 199.07532[M+H-2CO]^+^, 181.06461[M+H-2CO-H_2_O]^+^, 137.02327[M+H-C_8_H_6_O]^+^ |
| 44 |  |  |  |  | [M-H]^-^ | 253.05063 | 253.04974 | -3.525 | 253.05014[M-H]^-^, 178.99780[M-H-2CO-H_2_O]^-^, 135.00737[M-H-C_8_H_6_O]^-^, 107.01254[M-H-C_8_H_6_O-CO]^-^ |
| 45 | 11.17 | 3,3',4',5,7-pentamethoxyflavone | Flavonoids | C_20_H_20_O_7_ | [M-H]^-^ | 371.11363 | 371.1124 | -0.349 | 371.11301[M-H]^-^, 353.10257[M-H-H_2_O]^-^, 323.05545[M-OCH_3_-H_2_O]^-^, 267.06580[M-OCH_3_-H_2_O-CO]^-^, 191.03389[M-H-C_9_H_8_O_4_]^-^ |
| 46 | 11.47 | Luteolin | Flavonoids | C_15_H_10_O_6_ | [M+H]^+^ | 287.05501 | 287.05487 | -0.503 | 287.05487[M+H]^+^, 269.04410[M+H-H_2_O]^+^, 241.04887[M+H-CO-H_2_O]^+^, 153.01810[M+H-C_8_H_6_O_2_]^+^ |
| 46 | 11.48 |  |  |  | [M-H]^-^ | 285.04046 | 285.03973 | -2.566 | 285.04001[M-H]^-^, 267.02921[M-H-H_2_O]^-^, 241.05003[M-H-CO_2_]^-^, 151.00238[M-H-C_8_H_6_O_2_]^-^, 133.02814[M-H-C_7_H_7_O_4_]^-^ |
| 47 | 11.79 | 6-O-trans-cinnamoylphlorigidoside b | Terpenoids | C_29_H_36_O_13_ | [M-H]^-^ | 591.20831 | 591.20691 | -0.520 | 591.20691[M-H]^-^, 179.03348[Glc]^-^, 161.02296[Glc-H_2_O]^-^, 133.02797[Glc-H_2_O-CO]^-^ |
| 48 | 11.85 | Acacetin-7-O-β-glucuronide | Flavonoids | C_22_H_20_O_11_ | [M-H]^-^ | 459.09328 | 459.09229 | -2.166 | 459.09229[M-H]^-^, 268.03693[M-H-GlcA-CH_3_]^-^, 240.04141[M-H-GlcA-CH_3_-CO]^-^ |
| 49 | 12.06 | 5,7,3′,4′-tetrahydroxy-3-methoxyflavanone | Flavonoids | C_16_H_12_O_7_ | [M-H]^-^ | 315.05103 | 315.05032 | -2.241 | 315.05032[M-H]^-^, 300.02698[M-H-CH_3_]^-^, 271.02438[M-H-CO_2_]^-^, 227.03426[M-H-2CO_2_]^-^ |
| 49 | 12.06 |  |  |  | [M+H]^+^ | 317.06558 | 317.06543 | -0.471 | 317.06552[M+H]^+^, 302.04123[M+H-CH_3_]^+^, 274.04666[M+H-CO-CH_3_]^+^, 163.03905[M+H-C_7_H_6_O_4_]^+^, 135.04411[M+H-C_7_H_6_O_4_-CO]^+^ |
| 50 | 12.24 | Benzyl-4'- hydroxy-benzoyl-3'-O-β-D-glucopyranoside | Organic acids | C_20_H_22_O_9_ | [M-H]^-^ | 405.11911 | 405.11813 | -2.408 | 405.11813[M-H]^-^, 243.06612[M-H-Glc]^-^ |
| 51 | 13.04 | Abieta-8,11,13,15-tetraen-18-oic acid | Terpenoids | C_20_H_26_O_2_ | [M+H]^+^ | 299.20056 | 299.20035 | -0.691 | 299.20035[M+H]^+^, 284.17709[M+H-CH_3_]^+^, 269.15384[M+H-2CH_3_]^+^, 255.13808[M+H-CO_2_]^+^ |
| 52 | 13.06 | 5,7,4′-trihydroxy-3′-methoxyflavanone | Flavonoids | C_16_H_12_O_6_ | [M-H]^-^ | 299.05611 | 299.05569 | -1.410 | 299.05569[M-H]^-^, 284.03223[M-H-CH_3_]^-^, 256.03723[M-H-CH_3_-CO]^-^, 151.00243[M-H-C_9_H_8_O_2_]^-^ |
| 52 | 13.07 |  |  |  | [M+H]^+^ | 301.07066 | 301.07034 | -1.078 | 301.07034[M+H]^+^, 286.04684[M+H-CH_3_]^+^, 258.05197[M+H-CO-CH_3_]^+^, 153.01817[M+H-C_9_H_8_O_2_]^+^ |
| 53 | 13.17 | 5,7,2',6'-tetrahydroxyflavone | Flavonoids | C_15_H_10_O_6_ | [M-H]^-^ | 285.04046 | 285.03967 | -2.776 | 285.03986[M-H]^-^, 257.04507[M-H-CO]^-^, 151.00243[M-H-C_8_H_6_O_2_]^-^, 133.02817[M-H-C_7_H_7_O_4_]^-^ |
| 53 | 13.18 |  |  |  | [M+H]^+^ | 287.05501 | 287.05487 | -0.503 | 287.05499[M+H]^+^, 153.01810[M+H-C_8_H_6_O_2_]^+^ |
| 54 | 13.27 | 2α,3β,19α,23-tetrahydroxy-12-ene-28-oleanolic acid | Terpenoids | C_30_H_48_O_6_ | [M-H]^-^ | 503.33781 | 503.3367 | -0.031 | 503.33737[M-H]^-^, 401.26907[M-H-C_5_H_10_O_2_]^-^, 369.27930[M-H-C_5_H_10_O_2_-CH_3_OH]^-^, 351.26883[M-H-C_5_H_10_O_2_-CH_3_OH-H_2_O]^-^ |
| 55 | 13.72 | Cetraric acid | Organic acids | C_20_H_18_O_9_ | [M-H]^-^ | 401.08781 | 401.08667 | -0.096 | 401.08710[M-H]^-^, 357.05984[M-H-CO_2_]^-^, 313.07089[M-H-2CO_2_]^-^, 225.05475[M-H-C_9_H_4_O_4_]^-^, 181.06447[M-H-C_9_H_4_O_4_-CO_2_]^-^, 121.02795[M-HM-H-C_9_H_4_O_4_-CO_2_-C_3_H_8_O]^-^ |
| 56 | 13.77 | 8α,9α,13α,14α-diepoxyabietan-18-oic acid | Terpenoids | C_20_H_30_O_4_ | [M-H]^-^ | 333.20713 | 333.20627 | -2.589 | 333.20596[M-H]^-^, 303.19562[M-H-2CH_3_]^-^ |
| 57 | 14.04 | 7,8-epoxy-1(12)-caryophyllene-9β-ol | Terpenoids | C_15_H_24_O_2_ | [M+H]^+^ | 237.18491 | 237.18475 | -0.660 | 237.18475[M+H]^+^, 219.17442[M+H-H_2_O]^+^, 201.16348[M+H-2H_2_O]^+^, 194.08136[M+H-C_3_H_7_]^+^ |
| 58 | 14.46 | Isorhamnetin | Flavonoids | C_16_H_12_O_7_ | [M+H]^+^ | 317.06558 | 317.06543 | -0.471 | 317.06552[M+H]^+^, 302.04245[M+H-CH_3_]^+^, 274.04706[M+H-CO-CH_3_]^+^, 153.01843[M+H-C_9_H_8_O_3_]^+^ |
| 58 | 14.49 |  |  |  | [M-H]^-^ | 315.05103 | 315.15952 | -2.336 | 315.05060[M-H]^-^, 300.02692[M-H-CH_3_]^-^, 271.02451[M-H-CO_2_]^-^, 243.03014[M-H-CO_2_-CO]^-^, 151.00249[M-H-C_9_H_8_O_3_]^-^ |
| 59 | 15.04 | Rhamnazin | Flavonoids | C_17_H_14_O_7_ | [M-H]^-^ | 329.06668 | 329.0657 | -2.966 | 329.06570[M-H]^-^, 314.04236[M-H-CH_3_]^-^, 299.01889[M-H-2CH_3_]^-^, 271.02402[M-H-2CH_3_-CO]^-^, 243.02927[M-H-2CH_3_-2CO]^-^, 199.03885[M-H-2CH_3_-2CO-CO_2_]^-^, 151.00163[M-H-C_9_H_6_O_4_]^-^ |
| 60 | 15.62 | 2α,3β,22β,23-tetrahydroxyursolic-12-en-28-oic acid | Terpenoids | C_30_H_48_O_6_ | [M-H]^-^ | 503.33781 | 503.33682 | -2.150 | 503.33737[M-H]^-^, 485.32672[M-H-H_2_O]^-^, 457.33182[M-H-H_2_O-CO]^-^, 441.33676[M-H-H_2_O-CO_2_]^-^ |
| 61 | 15.66 | Callicarpone | Terpenoids | C_20_H_28_O_4_ | [M-H]^-^ | 331.19148 | 331.19061 | -2.635 | 331.19104[M-H]^-^, 316.16739[M-H-CH_3_]^-^, 299.16467[M-H-CH_2_-H_2_O]^-^ |
| 62 | 15.77 | Accacetin | Flavonoids | C_16_H_12_O_5_ | [M-H]^-^ | 283.0612 | 283.06033 | -3.062 | 283.06033[M-H]^-^, 268.03690[M-H-CH_3_]^-^, 240.04190[M-H-CH_3_-CO]^-^, 151.00241[M-H-C_9_H_8_O]^-^, 107.01234[M-H-C_9_H_8_O-CO_2_]^-^ |
| 63 | 17.02 | CH_3_O-pectolinarigenin | Flavonoids | C_18_H_16_O_7_ | [M-H]^-^ | 343.08233 | 343.0813 | -2.991 | 343.08176[M-H]^-^, 328.05798[M-H-CH_3_]^-^, 313.03455[M-H-2CH_3_]^-^, 285.03989[M-H-2CH_3_-CO]^-^, 226.02611[M-H-3CH_3_-CO-CO_2_]^-^ |
| 64 | 17.56 | Euscaphic acid | Terpenoids | C_30_H_48_O_5_ | [M-H]^-^ | 487.3429 | 487.34152 | -2.827 | 487.34210[M-H]^-^, 469.33136[M-H-H_2_O]^-^, 393.31442[M-H-H_2_O-HCOOH-2CH_3_]^-^ |
| 65 | 17.85 | Apigenin | Flavonoids | C_15_H_10_O_5_ | [M-H]^-^ | 269.04555 | 269.0448 | -2.775 | 269.04510[M-H]^-^, 241.05011[M-H-CO]^-^, 197.05991[M-H-CO-CO_2_]^-^, 169.06448[M-H-2CO-CO_2_]^-^, 151.00269[M-HC_8_H_6_O]^-^ |
| 66 | 18.43 | 7β-hydroxy dehydroabietic acid | Terpenoids | C_20_H_28_O_3_ | [M-H]^-^ | 315.19657 | 315.19583 | -2.341 | 315.19598[M-H]^-^, 300.02716[M-H-CH_3_]^-^ |
| 67 | 19.12 | Pentandralactone | Terpenoids | C_20_H_28_O_4_ | [M-H]^-^ | 331.19148 | 331.19067 | -2.454 | 331.19104[M-H]^-^, 313.18051[M-H-H_2_O]^-^, 287.16483[M-H-CO_2_]^-^ |
| 68 | 19.62 | 16,17-dihydroxy-3-oxophyllocladane | Terpenoids | C_20_H_30_O_3_ | [M-H]^-^ | 317.21222 | 317.21118 | -3.178 | 317.21118[M-H]^-^, 287.20087[M-H-CH_2_O]^-^ |
