# Supplementary material for: Anti-inflammatory properties and characterization of water extracts obtained from Callicarpa kwangtungensis Chun using in vitro and in vivo rat models
Source: Sci Rep. 2024 May 14;14:11047. doi: 10.1038/s41598-024-61892-9 (PMC11094131; doi:10.1038/s41598-024-61892-9)
Supplement: Supplementary file 19 — Supplementary Information 19. [file 41598_2024_61892_MOESM19_ESM.docx]

**Table 2**

Identification of 31 migrating constituents absorbed into serum by UHPLC-Q Exactive Orbitrap MS.

| No. | T_R_ (min) | Identification | Category | Formula | Ion mode | Theo. Mass  (m/z) | Obser. Mass (m/z) | Error  (ppm) | MS/MS fragments (m/z) |
| --- | --- | --- | --- | --- | --- | --- | --- | --- | --- |
| 1 | 4.40 | 3,4,5-trimethoxyphenyl-β-D-glucopyranoside | Phenols | C_15_H_22_O_9_ | [M+H]^+^ | 347.13366 | 347.1315 | -2.159 | 347.13150[M+H]^+^, 285.13098[M+H-2OCH_3_]^+^ |
| 2 | 5.51 | Forsythoside E | Phenylethanoid glycosides | C_20_H_30_O_12_ | [M-H]^-^ | 461.16645 | 461.16504 | -1.409 | 461.16504[M-H]^-^, 315.10870[M-H-Rha]^-^, 153.05437[phenethanol]^-^, 135.04364[phenethanol-H_2_O]^-^ |
| 3 | 5.78 | Peiioside A | Organic acids | C_26_H_36_O_17_ | [M-H]^-^ | 619.18797 | 619.18524 | -2.733 | 619.18634[M-H]^-^, 179.03365[caffeic acid-H]^-^, 161.02303[caffeic acid-H-H_2_O]^-^, 135.04367[caffeic acid-H-CO_2_]^-^ |
| 4 | 6.56 | 3,4-dihydroxybenzoic acid | Organic acids | C_7_H_6_O_4_ | [M-H]^-^ | 153.01933 | 153.01791 | -1.422 | 153.01788[M-H]^-^, 109.02794[M-H-CO_2_]^-^ |
| 5 | 6.73 | Caffeic acid | Organic acids | C_9_H_8_O_4_ | [M-H]^-^ | 179.03498 | 179.0336 | -1.382 | 179.03362[M-H]^-^, 135.04366[M-H-CO_2_]^-^ |
| 6 | 6.79 | 4-hydroxy cinnamic aicd | Organic acids | C_9_H_8_O_3_ | [M-H]^-^ | 163.04007 | 163.0387 | -1.367 | 163.03865[M-H]^-^, 145.02817[M-H-H_2_O]^-^, 135.04369[M-H-CO]^-^, 119.04860[M-H-CO_2_]^-^ |
| 7 | 7.18 | Tuberonic acid glucoside | Organic acids | C_18_H_28_O_9_ | [M-H]^-^ | 387.16606 | 387.16498 | -1.076 | 387.16525[M-H]^-^, 207.10179[M-H-Glc-H_2_O]^-^, 163.11159[M-H-Glc-H_2_O-CO_2_]^-^ |
| 8 | 7.45 | Melilotoside | Organic acids | C_15_H_18_O_8_ | [M-H]^-^ | 325.09289 | 325.09195 | -0.941 | 325.09207[M-H]^-^, 179.05475[Glc]^-^, 163.03868[M-H-Glc]^-^, 145.02786[M-H-Glc-H_2_O]^-^ |
| 9 | 7.80 | Echinacoside | Phenylethanoid glycosides | C_35_H_46_O_20_ | [M-H]^-^ | 785.25097 | 785.25122 | 0.253 | 785.25122[M-H]^-^, 623.20190[M-H-caffeoyl]^-^, 461.16641[M-H-caffeoyl-Glc]^-^, 315.10880[M-H-caffeoyl-Glc-Rha]^-^, 179.03473[caffeic acid]^-^, 161.02428[caffeic acid-H_2_O]^-^, 135.04503[caffeic acid-CO_2_]^-^, 153.05525[phenethanol]^-^ |
| 10 | 7.82 | β-OH-poliumoside | Phenylethanoid glycosides | C_35_H_46_O_20_ | [M-H]^-^ | 785.25097 | 785.24884 | -2.127 | 785.25000[M-H]^-^, 179.03380[caffeic acid]-, 161.02316[caffeic acid-H_2_O]^-^ |
| 11 | 8.45 | Samioside | Phenylethanoid glycosides | C_34_H_44_O_19_ | [M-H]^-^ | 755.2404 | 755.23877 | -1.632 | 755.23816[M-H]^-^, 623.19830[M-H-Api]^-^, 593.20947[M-H-caffeoyl]^-^, 447.14832[M-H-caffeoyl-Rha]^-^, 179.03371[caffeic acid]-, 161.02295[caffeic acid-H_2_O]^-^ |
| 12 | 8.66 | 5-methylisophthalic acid monomethyl ester | Organic acids | C_10_H_10_O_4_ | [M-H]^-^ | 193.05063 | 193.04933 | -1.302 | 193.04945[M-H]^-^, 178.02612[M-H-CH_3_]^-^, 149.05951[M-H-CO_2_]^-^ |
| 13 | 8.83 | Acteoside | Phenylethanoid glycosides | C_29_H_36_O_15_ | [M-H]^-^ | 623.19814 | 623.19666 | -1.483 | 623.19696[M-H]^-^, 461.16580[M-H-caffeoyl]^-^, 315.10794[M-H-caffeoyl-Rha]^-^, 179.03384[caffeic acid]^-^, 161.02310[caffeic acid-H_2_O]^-^, 135.04375[caffeic acid-CO_2_]^-^ |
| 14 | 8.87 | Poliumoside | Phenylethanoid glycosides | C_35_H_46_O_19_ | [M-H]^-^ | 769.25605 | 769.25482 | -1.232 | 769.25482[M-H]^-^, 607.22351[M-H-caffeoyl]^-^, 461.16376[M-H-caffeoyl-Rha]^-^, 179.03383[caffeic acid]^-^, 161.02319[caffeic acid-H_2_O]^-^, 135.04385[caffeic acid-CO_2_]^-^ |
| 15 | 8.89 | Forsythoside B | Phenylethanoid glycosides | C_34_H_44_O_19_ | [M-H]^-^ | 755.2404 | 755.23834 | -2.062 | 755.23834[M-H]^-^, 593.20703[M-H-caffeoyl]^-^, 461.16846[M-H-caffeoyl-Api]^-^, 315.10757[M-H-caffeoyl-Api-Rha]^-^, 179.03371[caffeic acid-H]^-^, 161.02303[caffeic acid-H-H_2_O]^-^ |
| 16 | 9.08 | Alyssonoside | Phenylethanoid glycosides | C_35_H_46_O_19_ | [M-H]^-^ | 769.25605 | 769.25482 | -1.232 | 769.25482[M-H]^-^, 593.20795[M-H-feruloyl]^-^, 461.16589[M-H-feruloyl-Api]^-^, 175.03886[feruloyl-H_2_O]^-^, 161.02309[feruloyl-CH_3_OH]^-^ |
| 17 | 9.21 | Isoacteoside | Phenylethanoid glycosides | C_29_H_36_O_15_ | [M-H]^-^ | 623.19814 | 623.19635 | -1.793 | 623.19708[M-H]^-^, 461.16574[M-H-caffeoyl]^-^, 315.10867[M-H-caffeoyl-Rha]^-^, 179.03374[caffeic acid]^-^, 161.02313[caffeic acid-H_2_O]^-^, 135.04373[caffeic acid-CO_2_]^-^ |
| 18 | 9.49 | Longissimoside B | Phenylethanoid glycosides | C_36_H_48_O_19_ | [M-H]^-^ | 783.2717 | 783.26971 | -1.992 | 783.27008[M-H]^-^, 607.22333[M-H-feruloyl]^-^, 461.16553[M-H-feruloyl-Rha]^-^, 193.04951[feruloyl]^-^, 175.03883[feruloyl-H_2_O]^-^ |
| 19 | 9.66 | Apigenin-7-glucuronide | Flavonoids | C_21_H_18_O_11_ | [M-H]^-^ | 445.07763 | 445.07599 | -1.645 | 445.07648[M-H]^-^, 269.04477[M-H-GlcA]^-^, 225.05428[M-H-GlcA-CO_2_]^-^ |
| 19 | 9.66 | Apigenin-7-glucuronide | Flavonoids | C_21_H_18_O_11_ | [M+H]^+^ | 447.09219 | 447.09158 | -0.608 | 447.09219[M+H]^+^, 271.05978[M+H-GlcA]^+^, 153.01811[M+H-GlcA-C_8_H_6_O]^+^ |
| 20 | 10.08 | Chrysoeriol-7-O-β-D-glucopyranoside | Flavonoids | C_22_H_22_O_11_ | [M-H]^-^ | 461.10893 | 461.10547 | -3.465 | 461.10834[M-H]^-^, 446.08459[M-H-CH_3_]^-^, 298.04758[M-H-Glc]^-^, 283.02429[M-H-Glc-CH_3_]^-^, 255.02917[M-H-Glc-CH_3_-CO]^-^ |
| 21 | 10.21 | Ferulic acid | Organic acids | C_10_H_10_O_4_ | [M-H]^-^ | 193.05063 | 193.04959 | -1.042 | 193.04959[M-H]^-^, 178.02618[M-H-CH_3_]^-^, 149.05936[M-H-CO_2_]^-^ |
| 22 | 10.21 | O-(methoxycarbonyl) phenylacetic acid | Organic acids | C_10_H_10_O_4_ | [M-H]^-^ | 193.05063 | 193.04959 | -1.042 | 193.04959[M-H]^-^, 178.02623[M-H-CH_3_]^-^ |
| 23 | 10.93 | Daidzein | Flavonoids | C_15_H_10_O_4_ | [M-H]^-^ | 253.05063 | 253.05014 | -0.492 | 253.05014[M-H]^-^, 178.99780[M-H-2CO-H_2_O]^-^, 135.00737[M-H-C_8_H_6_O]^-^, 107.01254[M-H-C_8_H_6_O-CO]^-^ |
| 23 | 10.93 | Daidzein | Flavonoids | C_15_H_10_O_4_ | [M+H]^+^ | 255.06519 | 255.065 | -0.185 | 255.06499[M+H]^+^, 227.07013[M+H-CO]^+^, 199.07532[M+H-2CO]^+^, 181.06461[M+H-2CO-H_2_O]^+^, 137.02327[M+H-C_8_H_6_O]^+^ |
| 24 | 11.24 | 3,3',4',5,7-pentamethoxyflavone | Flavonoids | C_20_H_20_O_7_ | [M-H]^-^ | 371.11363 | 371.11301 | 0.481 | 371.11301[M-H]^-^, 353.10257[M-H-H_2_O]^-^, 323.05545[M-OCH_3_-H_2_O]^-^, 267.06580[M-OCH_3_-H_2_O-CO]^-^, 191.03389[M-H-C_9_H_8_O_4_]^-^ |
| 25 | 13.02 | 5,7,4′-trihydroxy-3′-methoxyflavanone | Flavonoids | C_16_H_12_O_6_ | [M-H]^-^ | 299.05611 | 299.05569 | -0.421 | 299.05569[M-H]^-^, 284.03223[M-H-CH_3_]^-^, 256.03723[M-H-CH_3_-CO]^-^, 151.00243[M-H-C_9_H_8_O_2_]^-^ |
| 26 | 13.27 | 2α,3β,19α,23-tetrahydroxy-12-ene-28-oleanolic acid | Terpenoids | C_30_H_48_O_6_ | [M-H]^-^ | 503.33781 | 503.33737 | -0.442 | 503.33737[M-H]^-^, 401.26907[M-H-C_5_H_10_O_2_]^-^, 369.27930[M-H-C_5_H_10_O_2_-CH_3_OH]^-^, 351.26883[M-H-C_5_H_10_O_2_-CH_3_OH-H_2_O]^-^ |
| 27 | 15.59 | 2α,3β,22β,23-tetrahydroxyursolic-12-en-28-oic acid | Terpenoids | C_30_H_48_O_6_ | [M-H]^-^ | 503.33781 | 503.33737 | -0.442 | 503.33737[M-H]^-^, 485.32672[M-H-H_2_O]^-^, 457.33182[M-H-H_2_O-CO]^-^, 441.33676[M-H-H_2_O-CO_2_]^-^ |
| 28 | 15.61 | Callicarpone | Terpenoids | C_20_H_28_O_4_ | [M-H]^-^ | 331.19148 | 331.19104 | -0.443 | 331.19104[M-H]^-^, 316.16739[M-H-CH_3_]^-^, 299.16467[M-H-CH_2_-H_2_O]^-^ |
| 29 | 17.78 | Apigenin | Flavonoids | C_15_H_10_O_5_ | [M-H]^-^ | 269.04555 | 269.0451 | -0.447 | 269.04510[M-H]^-^, 241.05011[M-H-CO]^-^, 197.05991[M-H-CO-CO_2_]^-^, 169.06448[M-H-2CO-CO_2_]^-^, 151.00269[M-HC_8_H_6_O]^-^ |
| 30 | 19.12 | Pentandralactone | Terpenoids | C_20_H_28_O_4_ | [M-H]^-^ | 331.19148 | 331.19104 | -0.443 | 331.19104[M-H]^-^, 313.18051[M-H-H_2_O]^-^, 287.16483[M-H-CO_2_]^-^ |
| 31 | 19.64 | 16,17-dihydroxy-3-oxophyllocladane | Terpenoids | C_20_H_30_O_3_ | [M-H]^-^ | 317.21222 | 317.21118 | -1.038 | 317.21118[M-H]^-^, 287.20087[M-H-CH_2_O]^-^ |
